# Selective immunocapture reveals neoplastic human mast cells secrete distinct microvesicle‐ and exosome‐like populations of KIT‐containing extracellular vesicles

**DOI:** 10.1002/jev2.12272

**Published:** 2022-10-14

**Authors:** Annika Pfeiffer, Jennifer D. Petersen, Guido H. Falduto, David Eric Anderson, Joshua Zimmerberg, Dean D. Metcalfe, Ana Olivera

**Affiliations:** ^1^ Mast Cell Biology Section Laboratory of Allergic Diseases National Institute of Allergy and Infectious Diseases National Institutes of Health Bethesda Maryland USA; ^2^ Section on Integrative Biophysics Division of Basic and Translational Biophysics Eunice Kennedy Shriver National Institute of Child Health and Human Development National Institutes of Health Bethesda Maryland USA; ^3^ Advanced Mass Spectrometry Core Facility National Institute of Diabetes and Digestive and Kidney Diseases National Institutes of Health Bethesda Maryland USA

**Keywords:** exosomes, extracellular vesicles, immunocapture, KIT, mast cell, microvesicles

## Abstract

Activating mutations in the receptor KIT promote the dysregulated proliferation of human mast cells (huMCs). The resulting neoplastic huMCs secrete extracellular vesicles (EVs) that can transfer oncogenic KIT among other cargo into recipient cells. Despite potential contributions to diseases, KIT‐containing EVs have not been thoroughly investigated. Here, we isolated and characterized KIT‐EV subpopulations released by neoplastic huMCs using an immunocapture approach that selectively isolates EVs containing KIT in its proper topology. Immunocapture of EVs on KIT antibody‐coated electron microscopy (EM) affinity grids allowed to assess the morphology and size of KIT‐EVs. Immunoblot analysis demonstrated KIT‐EVs have a distinct protein profile from KIT‐depleted EVs, contain exosome and microvesicle markers, and are separated into these subtypes by ultracentrifugation. Cell treatment with sphingomyelinase inhibitors shifted the protein content among KIT‐EV subtypes, suggesting different biogenesis routes. Proteomic analysis revealed huMC KIT‐EVs are enriched in proteins involved in signalling, immune responses, and cell migration, suggesting diverse biological functions, and indicated neoplastic huMCs disseminate KIT via shuttling in heterogeneous microvesicle‐ and exosome‐like EVs. Further, selective KIT‐immunocapture will enable the enrichment of specific huMC‐derived EVs from complex human biosamples and facilitate an understanding of their in vivo functions and potential to serve as biomarkers of specific biological pathologies.

## 1 INTRODUCTION

Extracellular vesicles (EVs) are secreted by all cell types, and since these nano‐sized vesicles surrounded by a lipid bilayer membrane carry proteins, nucleic acids and lipids, EVs are thought to play a functional role in cell‐to‐cell communication by transferring their molecular cargo from donor to recipient cells. Due to this capacity, EVs may become promising tools for diagnostics and therapeutics. Most EVs implicated in intercellular communication are categorized into two main subtypes based on size and the biogenesis route: exosomes are “small” EVs (50–150 nm) that form as intraluminal vesicles in the endosome to be released upon multivesicular body (MVB) fusion with the plasma membrane; microvesicles are “larger” EVs (100–>200 nm) that form and bud directly off the plasma membrane (Kalluri & LeBleu, 2020; Mathieu et al., 2019; van Niel et al., 2018). However, the classification of EVs is continuing to evolve as overlapping sizes among EV subtypes have been noted (Mathieu et al., 2019) and the list of vesicle types is expanding (Di Vizio et al., 2009; Nabhan et al., 2012; H. Zhang et al., 2018). Though there are yet no discriminatory markers, studies are starting to identify potential EV subtype‐specific protein markers (Jeppesen et al., 2019; Kowal et al., 2016; Kugeratski et al., 2021; Mathieu et al., 2021).

Moreover, the two major EV subtypes appear to further subdivide into heterogeneous subpopulations distinguished by their varying molecular cargo composition (Kowal et al., 2016). Reports that the release of EV subpopulations may be influenced by factors like cell activation (Groot Kormelink et al., 2016a; van der Vlist et al., 2012) or malignant transformation (Palma et al., 2012) suggest an emerging question as to whether subpopulations of EVs secreted by transformed or activated cells in pathological conditions contain distinct cargo that may convey specific biological functions and potentially serve as biomarkers for disease diagnosis, progression and/or response to specific therapeutic intervention (Boyiadzis & Whiteside, 2017). These aspects emphasize the need for further study and detailed characterization of EV subtypes and subpopulations released by single cell types.

The tyrosine kinase KIT (CD117) is a critical receptor for proliferation, survival, differentiation and homing of hematopoietic stem cells as well as human mast cells (huMCs), which retain KIT expression after differentiation and maturation. Mast cells are generally considered in the context of immune cells that mediate allergic inflammatory responses by releasing mediators contained in granules, generating lipid‐derived molecules, and secreting cytokines and chemokines after activation. In addition, mast cells are known to secrete EVs both in a resting and activated cell state (Carroll‐Portillo et al., 2012; Groot Kormelink et al., 2016; Liang et al., 2020; Molfetta et al., 2020; Raposo et al., 1997). EVs released by activated mast cells have been proposed to contribute to allergic reactions (Carroll‐Portillo et al., 2012; Molfetta et al., 2020) and contain the receptor KIT (Groot Kormelink et al., 2016; Liang et al., 2020). Moreover, EVs containing KIT are increased in the serum of patients with systemic mastocytosis (Kim et al., 2018), a clonal disease of the huMC compartment associated with KIT variants, and in the plasma of patients with CD34^+^ acute myeloid leukaemia (Boyiadzis & Whiteside, 2017; Hong et al., 2014; Szczepanski et al., 2011). We and others have also reported that KIT is shuttled from neoplastic huMC EVs into recipient cells in vitro and in vivo, affecting cell phenotypes and functions (Kim et al., 2018; Kim et al., 2021; Xiao et al., 2014). In this way, huMC‐derived KIT‐EVs may contribute to the pleiotropic clinical manifestations of mastocytosis, opening possibilities for investigating disease biomarkers (Falduto et al., 2020). Additionally, it has been reported that gastrointestinal stromal tumour (GIST) cells release EVs that similarly contain oncogenic KIT with tumour‐promoting activities and that KIT‐containing exosomes can be isolated from GIST cell lines and patient plasma (Atay et al., 2014, 2018). The potential biological impact of EVs in mastocytosis and other haematological disorders as well as in allergic and malignant diseases poses a functional interest in KIT‐containing EVs, and their further characterization is well warranted.

In this study, we thus investigated and characterized KIT‐containing EVs secreted by neoplastic huMCs. KIT‐EVs were successfully and efficiently isolated by selective immunocapture, depending on the topology of KIT in the EV membrane. The protein content of such isolated KIT‐EVs differed substantially from KIT‐depleted, CD9‐captured vesicles, underlining the heterogeneity among mast cell EVs. Furthermore, even within the KIT‐containing EVs, both microvesicle‐ and exosome‐like EVs separated by density and surface affinity into EV subtypes with distinct protein cargo. Proteomic analysis indicated that KIT‐EVs are enriched in proteins involved in signalling, immune responses, and cell migration, suggesting that KIT‐containing EVs have different biological functions. The application of this work to other cell models and for enriching KIT‐containing huMC‐EVs from complex biological samples is discussed.

## 2 MATERIALS AND METHODS

### 2.1 Cell culture

The neoplastic huMC lines HMC‐1.1 (*KIT* V560G) and HMC‐1.2 (*KIT* V560G, *KIT* D816V) were kindly provided by Dr. JH Butterfield (Mayo Clinic, Rochester, MN, USA) (Butterfield et al., 1988). HMC‐1 cells were maintained in a humidified incubator at 37°C and 5% CO_2_ in T75 flasks in Iscove's modified Dulbecco's medium (IMDM, Corning) supplemented with 2 mM L‐Glutamine, 100 IU/ml penicillin, 100 μg/ml streptomycin (Corning) and 10% (v/v) fetal bovine serum (FBS, Gibco, Thermo Fisher Scientific). Cells were passaged every 3–4 days based on cell density. The LAD2 huMC line was maintained in serum‐free StemPro‐34 media supplemented with StemPro‐34 nutrient supplement (Gibco), 2 mM L‐Glutamine, 100 IU/ml penicillin, 100 μg/ml streptomycin (Corning) and 100 ng/ml recombinant human SCF (R&D Systems), as described (Kirshenbaum et al., 2003). Hemi‐depletions were performed weekly. Cell counts and viability (using acridine orange/propidium iodide stain, Logos Biosystems) were assessed on a Luna‐FL automated brightfield and dual fluorescence cell counter (Logos Biosystems). Cell treatment with 10 μM GW4869 (5 mM stock solution in DMSO; Sigma‐Aldrich), DMSO (vehicle control; Sigma‐Aldrich) or 10 μM amitriptyline (10 mM stock solution in water; Sigma‐Aldrich) was performed for 16 h.

### 2.2 Isolation of EVs by polymer precipitation and differential ultracentrifugation

All EV isolation steps were carried out at 4°C or on ice and included cold filtered 1× PBS obtained by using a 0.22 μm PES membrane filter. For the isolation of EVs, a ratio of 10^6^ HMC‐1 cells/ml media were cultured in T175 flasks for 48–72 h in media containing EV‐depleted FBS (obtained by ultracentrifugation at 120,000 × *g* for 24 h, in an SW32‐Ti rotor [Beckman Coulter] and passaging through a 0.22 μm PES filter). Cell viability was determined to be >95% before isolating EVs from the media. HMC‐1 cell‐derived EVs released into the culture supernatant were isolated by ExoQuick‐TC solution (System Biosciences) for initial experiments. Collected media was cleared (3000 × *g*, 15 min, Thermo Scientific TX‐750 swinging bucket rotor), combined with ExoQuick‐TC solution (1 ml per 5 ml media) and incubated overnight at 4°C without rotation. EV pellets were obtained (1500 × *g*, 1 h, 4°C, TX‐750 swinging bucket rotor), pellet tops were carefully rinsed 3× with PBS (rinsing was performed without resuspension of the pellet), and finally resuspended in 50 μl of PBS by gently mixing on a shaker (300 rpm, 30 min, 4°C) (Eppendorf Thermo Mixer).

For immunocapture assays, EVs were precipitated by combining pre‐cleared culture supernatant (3000 × *g*, 15 min, TX‐750 swinging bucket rotor) with a stock solution of 32% PEG (MW 8000, Sigma‐Aldrich) prepared in 2 M filtered NaCl (Sigma‐Aldrich) to obtain a final concentration of 8% PEG. Precipitation and rinsing steps were identical to ExoQuick‐treated media described above. 8% PEG precipitates of fresh culture media alone (mock) did not contain any detectable EV markers (Figure S1A).

Alternatively, EVs were obtained from culture media by differential ultracentrifugation. Cells were first pelleted at 400 × *g* for 10 min (TX‐750 swinging bucket rotor) at 4°C. The supernatant was collected and sequentially subjected to centrifugation at 2000 × *g* (20 min, 4°C, TX‐750 swinging bucket rotor) to sediment debris and apoptotic bodies, and then at 15,000 × *g* for 40 min at 4°C (Sorvall Legend XTR, Fiberlite F15‐8 × 50cy rotor, fixed angle) to pellet microvesicle‐like EVs (P15 EVs, large EVs). The P15 pellet was washed in PBS and re‐pelleted (15,000 × *g*, 40 min, 4°C). The supernatant after the first 15,000 × *g* spin was centrifuged at 120,000 × *g* (2 h, 4°C) in a SW40‐Ti or SW32‐Ti rotor (OptimaXE‐90, Beckman Coulter) to obtain a pellet of exosome‐like EVs (P120 EVs, small EVs). The P15 and P120 pellets were resuspended in PBS by gently mixing on a shaker (300 rpm, 30 min, 4°C) and immediately processed or stored at ‐80°C.

EVs were lysed in RIPA buffer (Cell Signalling Technology) supplemented with protease (complete mini, EDTA‐free, Roche) and phosphatase inhibitors (phosSTOP, Roche) (20 min on ice with occasional vortexing). The protein content in the EV preparations was determined with a bicinchoninic acid (BCA) protein assay (Pierce, Thermo Scientific).

### 2.3 Isolation of EVs by size exclusion chromatography (SEC)

Cell culture media was pre‐cleared (400 × *g* 10 min, 2000 × *g* 20 min; TX‐750 swinging bucket rotor), concentrated using an Amicon Ultra‐15 centrifugal filter unit (10 kDa cutoff, Millipore) and added to a qEVoriginal/70 nm size exclusion column (IZON). EVs were isolated and eluted off the column following the manufacturer's instructions. In brief, the column was washed and equilibrated with 10 ml freshly prepared filtered PBS before adding 0.5 ml of concentrated media containing EVs. The void volume (3 ml; fractions 1–6) was discarded, and the collected pooled EV fraction (2.5 ml; fractions 7–11) was analyzed by NTA, as described below, and immunoblotting. Where needed, the eluted EV fraction was concentrated in Amicon Ultra‐0.5 centrifugal filter units (10 kDa cutoff, Millipore). For the immunocapture of KIT‐EVs from SEC‐isolated EVs, SEC fractions seven to nine (1.5 ml), containing the majority of EVs, were pooled, and directly combined with capturing beads to avoid EV loss during filter concentration.

### 2.4 Isolation of LAD2 huMC‐derived EVs

LAD2 cells were cultured overnight in cytokine‐free StemPro‐34 media (with nutrient supplement). The next day, cells were washed with HEPES buffer containing 0.04% BSA (the buffer was filtered through a 0.22 μm PES membrane filter after the addition of BSA) (Kuehn et al., 2010). 15–20 × 10^6^ cells were incubated at 37°C (without CO_2_) in HEPES‐BSA buffer with or without rhSCF (100 ng/ml). Supernatants and cells were collected after 2 h. EVs released into the supernatant were isolated by differential ultracentrifugation as described above.

### 2.5 Immunocapture of EV subpopulations on beads

Antibody‐conjugated magnetic dynabeads (commercial CD9, CD81, and CD63 pre‐conjugated beads, Invitrogen, Thermo Fisher Scientific) were washed 3× in 0.5 ml filtered PBS (bead recovery was performed on a magnetic rack) and incubated with equal numbers of PEG‐precipitated EVs, EVs obtained by differential ultracentrifugation or SEC‐purified EVs (1‐3 × 10^10^ vesicles/immunocapture) in a total volume of 0.2 ml or a maximal total SEC eluate volume of 1.5 ml under rotation and tilting overnight at 4°C.

For the immunocapture of KIT‐containing EVs, 3 μg of KIT antibody (R&D Systems, AF332, directed against the N‐terminal domain of KIT [Gln26‐Thr520], goat; Santa Cruz, sc‐13508, directed against full‐length KIT, mouse; or R&D Systems, AF3267, directed against a part in the C‐terminus of KIT [Asp716‐Pro726], rabbit) were conjugated to 30 μl of PBS‐washed magnetic dynabeads slurry (protein G, Invitrogen, Thermo Fisher Scientific) under rotation and tilting for 1 h at 4°C, followed by incubation with PEG‐precipitated, SEC‐isolated, P15 or P120 EVs under rotation and tilting overnight at 4°C. For KIT topology assays, PEG‐EVs were lysed or not with 0.1% Triton X‐100 detergent for 10 min at room temperature prior to the KIT‐EV immunocapture. EVs incubated with unconjugated beads or beads conjugated to 3 μg isotype control antibodies (goat anti‐HA, Novus Biologicals NB600‐362; rabbit anti‐FLAG, Invitrogen 710662; mouse anti‐V5, Invitrogen 46–0707) served as negative binding controls. Beads bound to immunocaptured EVs were washed at least 3× with cold, filtered PBS using a magnetic rack on ice and transferred to clean microtubes with the last washing step. Where indicated, the flow‐through containing unbound EVs was exposed to CD9 or CD81 pre‐conjugated beads for the isolation of CD9 or CD81 positive/KIT‐depleted EVs. Where indicated, unbound EVs in the flow‐through were pelleted by ultracentrifugation (120,000 × *g* for 1 h at 4°C in a SW55‐Ti rotor) to demonstrate the immunocapture efficiency.

For EV lysis and elution, beads were suspended in 10 μl of RIPA buffer (Cell Signalling Technology) supplemented with protease (complete mini, EDTA‐free, Roche) and phosphatase (phosSTOP, Roche) inhibitors, sonicated in a water bath sonicator for 30 sec and incubated on ice for 20 min, followed by the addition of 10 μl 2× LDS sample buffer (Invitrogen, Thermo Fisher Scientific) and heating at 40°C for 10 min. Extracted proteins were separated from the beads with a magnetic rack before loading the total elution volume into NuPAGE gels for analysis.

### 2.6 Density gradients on EV pellets

To obtain high‐resolution iodixanol gradients, P15 and P120 EV pellets were resuspended in 1 ml 60% iodixanol (OptiPrep, Sigma‐Aldrich) and bottom‐loaded in ultraclear ultracentrifugation tubes (Beckman Coulter). Descending concentrations of 2 ml iodixanol solutions (36%, 30%, 24%, 18%, and 12%), prepared in filtered PBS, were sequentially layered on top and the gradient was subjected to ultracentrifugation at 100,000 × *g* with slow acceleration and deceleration for 17 h at 4°C in a SW40‐Ti rotor (Beckman Coulter). Twelve 1 ml fractions containing EVs, separated based on density, were collected from the top. Each fraction was thoroughly mixed with 11 ml of filtered PBS and then the EVs were pelleted at 100,000 × *g* in a SW40‐Ti rotor for 2 h at 4°C. The refractive index of each fraction was measured on a digital refractometer (model DR301‐95, Kruess) and related to the density of the iodixanol solution (https://www.optiprep.com/V01.pdf). Final EV pellets from each fraction were resuspended in 25 μl filtered PBS.

### 2.7 Nanoparticle tracking analysis (NTA) of EVs

For every experiment (*n* > 3), particle size distribution and concentration of isolated EVs were determined on a NanoSight NS300 instrument (Malvern Panalytical). Diluted EV samples were injected into the laser chamber at a constant infusion rate by a syringe pump (Harvard Apparatus). Particles in the samples were captured at room temperature for 30 sec with five repetitions with a camera level of 10–12. NTA software (NTA 3.3 Dev Build 3.3.301) was used for recording, data processing and calculation of the concentration (particles/ml) and particle diameter (nm) for each repetition as well as the average of five dynamic measurements. The concentration of the EV samples was corrected by the dilution factor.

### 2.8 Spiking human plasma with cell culture EVs

To spike normal human plasma with HMC‐1.1‐derived EVs, EVs were first PEG‐precipitated from culture supernatant as described above. 5 μg of anti‐KIT (AF332, R&D Systems; goat) or isotype control anti‐HA (NB600‐362, Novus Biologicals; goat) antibodies were covalently coupled to dynabeads (M‐270 Epoxy) according to the manufacturer's instructions (Dynabeads Antibody Coupling Kit, Thermo Fisher Scientific). Human plasma stored at ‐80°C was thawed on ice, combined with 1× Halt protease and phosphatase inhibitor cocktail (Thermo Fisher Scientific), cleared at 2000 × *g* for 10 min at 4°C (TX‐750 swinging bucket rotor), and further incubated with empty PBS‐washed dynabeads under rotation for 30 min at 4°C to minimize unspecific binding of plasma components to the antibody‐conjugated beads. 1.2 × 10^10^ EVs were incubated in 500 μl pre‐cleared plasma for 1 h at 4°C on a shaker (400 rpm) followed by immunocapture as described above. Where indicated, HMC‐1.1 EVs were pre‐incubated with 2 ng/ml SCF (1 h at 4°C on a shaker at 400 rpm) prior to KIT immunocapture.

Plasma collection of healthy volunteers was conducted under protocol NIAID 09‐I‐0049 (NCT00806364) approved by the National Institute of Allergy and Infectious Diseases (NIAID) Institutional Review Board in compliance with the ethical regulations determined on the approved protocols by the NIH Institutional Review Board, and in agreement with the Declaration of Helsinki. All participants provided written informed consent.

### 2.9 Immunoblotting

PBS‐washed cell lysates were prepared on ice in RIPA buffer supplemented with protease and phosphatase inhibitors as described above for EV preparations. Detergent‐insoluble cellular material was pelleted at 13,000 rpm for 10 min (4°C). The protein concentration of lysed cell samples was measured by the BCA protein assay.

Lysed cell or EV preparations were combined with an appropriate volume of 4× NuPAGE LDS sample buffer (Invitrogen, Thermo Fisher Scientific) under non‐reducing conditions and heated at 40°C for 10 min. Equal protein amounts (typically 30 μg), equal numbers of vesicles or total immunocaptured EV eluates from the indicated starting material (see figure legends) were loaded and separated on NuPAGE Bis‐Tris 4%–12% gels (Thermo Fisher Scientific), followed by transfer to nitrocellulose membrane (either by wet transfer in a XCell II blot module (Thermo Fisher Scientific) at 35 V for 70 min or by dry transfer in a turbo transfer system (Biorad) at 25 V for 10 min). Membranes were air‐dried for 10 min and blocked in Odyssey blocking buffer (formulated in TBS; LI‐COR Biosciences) for 1 h at room temperature. The following primary antibodies were used at a dilution of 1:1000, unless stated otherwise, in blocking buffer and membranes were incubated overnight on a shaker at 4°C: rabbit anti‐syntenin‐1 (Abcam, ab133267), mouse anti‐CD81 (R&D Systems, MAB4615), rabbit anti‐annexin A1 (Abcam, ab214486; 1:2000), rabbit anti‐ALIX (Abcam, ab186429), mouse anti‐flotillin‐1 (BD Biosciences, 610821), rabbit anti‐TSG101 (Abcam, ab125011), rabbit anti‐CD9 (Abcam, ab92726), mouse anti‐calreticulin (BioVision, 3076–100), mouse anti‐CD63 (BD Biosciences, 556019), goat anti‐KIT (R&D Systems, AF332), mouse anti‐KIT (Santa Cruz, sc‐13508), rabbit anti‐KIT (R&D Systems, AF3267), rabbit anti‐phospho‐KIT Tyr823 (Invitrogen, 44–498G), rabbit anti‐HSP90 (Cell Signalling, 4877S), rabbit anti‐ARF6 (Abcam, ab226389), rabbit anti‐histone H3 (Cell Signalling, 9715S; 1:2000), mouse anti‐mast cell tryptase (Abcam, ab2378), mouse anti‐β‐actin (Sigma‐Aldrich, A5441; 1:10,000), rabbit anti‐SCF (Invitrogen, PA5‐20746). Membranes were rinsed with TBST (TBS with 0.1% Tween‐20) followed by three TBST washes for 5 min at room temperature. Secondary antibodies were donkey anti‐mouse IRDye680RD or IRDye800CW, donkey anti‐goat IRDye680RD or IRDye800CW, donkey anti‐rabbit IRDye680RD or IRDye800CW (LI‐COR Biosciences), used at a dilution of 1:20,000 in TBST/blocking buffer. Antibody‐probed membranes were washed three times with TBST, rinsed with TBS and imaged with a LI‐COR Odyssey CLx scanner. Analysis and quantification were performed using Image Studio Lite software (version 5.2, LI‐COR).

### 2.10 Sample preparation for SILAC‐based quantitative mass spectrometry of EVs

For SILAC labelling, IMDM deficient in both L‐lysine and L‐arginine (Thermo Fisher Scientific) was supplemented with dialyzed FBS (Thermo Fisher Scientific), 100 IU/ml penicillin and 100 μg/ml streptomycin (Corning). Both 0.798 mM L‐lysine HCl and 0.398 mM L‐arginine HCl were added to prepare “light” SILAC media. “Heavy” SILAC media was supplemented with 0.798 mM ^13^C_6_ L‐lysine 2xHCl (Cambridge Isotope Laboratories) and 0.398 mM ^13^C_6_ L‐arginine HCl (Silantes). To counteract the conversion of arginine to proline, the media was additionally supplemented with 200 mg/l L‐proline. Media was filtered through a 0.22 μm PES filter after preparation. HMC‐1.1 cells were passaged every 3 days in fresh “heavy” or “light” media to allow the metabolic incorporation of heavy isotope amino acids, which was confirmed by a cell check at passage 5.

For the isolation of EVs, cells were cultured for 72 h in “heavy” or “light” SILAC media supplemented with dialyzed EV‐depleted FBS (obtained by ultracentrifugation at 120,000 × *g*, 24 h, 4°C; SW40‐Ti rotor). EVs were isolated by SEC and immunocapturing of KIT‐EVs was performed as described above. The flow‐through, containing KIT‐depleted EVs, was subjected to CD9 immunocapturing (see Figure S4A). Alternatively, EVs were first pelleted at 15,000 × *g* and 120,000 × *g* to obtain P15 and P120 EVs, followed by SEC as a “cleaning” step and KIT immunocapture. All washing steps were performed with filtered PBS. Captured EV populations were eluted with 10 μl of 1% sodium dodecanoate under heating at 60°C for 20 min, after which “heavy” and “light” EV populations were combined prior to further processing for the mass spectrometry analysis. Both reciprocal pairs (H/L and L/H) were prepared and analyzed. The Pearson's correlation between duplicates (log2 ratios H/L and L/H) in the KIT‐EVs versus CD9/KIT‐depleted EVs pairs was *R* = 0.826 (*p* < 0.0001), and in the P15 vs P120 EVs comparison, *R* = 0.734 (*p* < 0.0001). A bio‐replicate match check of immunocaptured KIT‐EVs was also initially performed to establish empirically that the sample preparation was reproducible.

### 2.11 Proteomic analysis

The eluates were made 50 mM Tris and 5 mM DTT and heated at 90°C for 10 min. Next, the sample was made 15 mM 2‐chloroacetamide and incubated for 45 min under a foil cover. The sample was then incubated for 15 min with 10 mM beta‐mercaptoethanol to scavenge unreacted 2‐chloroacetamide and then diluted with 50 mM Tris to a final concentration of 0.25% dodecanoate. The samples were treated with 2.5 μg trypsin (Promega) overnight at room temperature. The next day, the sample was acidified by adding 1/10 volume of 10% formic acid and peptides extracted with ethyl acetate three times (Lin et al., 2013). After the third extraction, the aqueous phase was transferred to a fresh vial. 100 μl of 0.4% formic acid were added to the older vial and once the phase had re‐established, most of this solution was moved to the fresh vial as well. After settling the sample briefly at 900 × *g*, the sample was warmed to 50°C under a stream of dry nitrogen gas to blow off residual ethyl acetate. Then, the sample was applied to a serial stack comprising a C8 STAGE tip exiting into the top of a C18 STAGE tip (4 wide bore plugs each with significant overcapacity were used due to uncertainty about non‐peptide components competition) (Rappsilber et al., 2007) using a 200 × *g* centrifugal loading at 4°C. After washing the column stack with 3 × 150 μl of 1.6% formic acid 50 mM ammonium acetate columns were reversed in order, the centrifuge heated to 40°C, and then 100 μl of a warmed (50°C) solution of 0.4% formic acid/40% acetonitrile were applied and the eluate collected in a new vial at 400 × *g* for 5 min. This was followed by a similar volume of 0.4% formic acid/80% acetonitrile. Upon nitrogen dry down, these samples had significant physical residue, so pre‐purification was extended to strong cation exchange (SCX). Samples were taken up in 200 μl of 0.4% formic acid 40% acetonitrile and loaded onto a four‐core SCX stage tip at 200 × *g*. The tip was then washed with increasing organic solution (3 × 150 μl 0.4% formic acid, 80% acetonitrile, 3 × 150 μl acetonitrile). In the case of the KIT/CD9 experiments, peptides on the SCX column were eluted with exchange solution and analyzed as a single fraction sample. In the case of the P15/P120 experiments, we explored improving coverage by using step fractions from the SCX column as we had more confidence that this approach would not be detrimental due to sample dilution. Fractionation was as described (Kulak et al., 2014). Once the eluted samples were dried to near dryness with a stream of nitrogen gas, a further 20 μl of 50% acetonitrile was added and this was dried down to near dryness. Then, the sample (volume less than 5 μl) was diluted with 30 μl of 0.1% formic acid/4% acetonitrile and the sample briefly heated with agitation to 50°C, placed in a sanitation bath (in a foam float) for 5 min and then spun down briefly at 900 × *g* before submission to the queue of the LC/MS/MS system.

The LC/MS/MS system comprised a Thermo nLC‐1000 with after‐market modified Stator (C72‐1C76U) and Rotors (C72‐1C76U) from OEM VICI and a Thermo Lumos mass spectrometer. The system was configured for one‐column operation using a 50 cm Easy Spray column. Solution A was 0.1% formic acid and solution B was 0.1% formic acid ca. 93.75% acetonitrile (15/16ths). Columns were loaded at a system‐regulated pressure of 500 psi and then a separation gradient was run; 5% to 18% in 215 min, to 28% B in 30 min, to 95% B in 20 min, and a further 35 min at 95% to account for system delay. A 2100 V potential was applied through the Easy Spray source with a 200C ion transfer tube. The mass spectrometer was set up to collect a single high‐resolution spectrum (120K, 475–1500 M/Z, RF 30%, AGC 1E6, max injection 246 ms) and then spend up to 7 sec collecting lower resolution fragment spectra (CID/ion trap, quad isolated, 2 m/z window, 2E4 AGC target, 120 ms maximum injection time with all available parallelization allowed) with dynamic exclusion (two times, 20 sec, 20 sec).

Raw data were searched using MaxQuant v1.6.10.43 (Cox & Mann, 2008) using default settings against common contaminants and the human reference proteome (downloaded 11/07/2020). Label inversion was accommodated by switching the specification in the type panel (heavy forms of amino acid for light labels etc.). Enriched proteins were analyzed using the Perseus software v1.6.14.0 to determine significantly changed proteins (Table S3). Significantly changed proteins were submitted in ShinyGO v0.741 for gene ontology and KEGG pathway analysis (http://bioinformatics.sdstate.edu/go/). These analyses did not use normalized ratios because the amount of protein between compared EV groups was expected to be different. To confirm the conclusions, we also conducted 1D GO term analyses (Cox & Mann, 2012) using a normalization mode based on the median of the observed peptide ratios for each protein in the sample, an approach that minimizes the identification of changes due to differences in total protein between samples (Figure S5). Heatmaps of the normalized log2 ratios were generated using Morpheus (https://software.broadinstitute.org/morpheus/).

### 2.12 Negative stain electron microscopy (EM) of EVs immunocaptured on KIT antibody‐coated affinity grids

The optimal dilution of PEG‐precipitated EVs in PBS for negative stain EM was determined, so that individual EVs bound to the EM grid surface could be clearly resolved, unobscured by other EVs and entities present in the preparation. To perform negative stain EM, undiluted or EVs further diluted 1:10, 1:20 and 1:40 in filtered PBS (Gibco) were adhered to freshly glow discharged carbon and formvar coated 300 mesh gold EM grids (Electron Microscopy Sciences) by floating the grid on a 5–10 μl drop of EV preparation for 2 min. Grids were then rinsed three times with PBS by floating the grids on drops of PBS, rinsed twice with filtered water, and then placed on drops of 1% aqueous uranyl acetate negative stain solution (Electron Microscopy Sciences) for 60 sec. Finally, grids were blotted to dryness with filter paper and observed by transmission EM to select the optimal EV concentration.

To prepare KIT immunocapture affinity grids, grids were coated with a primary antibody to KIT, as described (Derrick, 1973; Yu et al., 2016), prior to adherence of EVs. This approach significantly reduced the non‐specific binding to the grid of KIT‐negative species present in the preparation, providing cleaner images for analysis (see Figure S3B,C). Grids (as described above) were incubated for 10 min on 10 μl drops of goat anti‐KIT antibody (R&D Systems, AF332) diluted to 20 mg/ml in PBS. Grids were rinsed twice with PBS, and then placed on drops of filtered blocking solution containing 2% BSA (Sigma) in PBS for 10 min. After blocking, grids were incubated on 5–10 μl drops of EV preparations at the preferred dilution for 5 min, allowing KIT‐EVs to selectively bind to the antibody‐coated grid surface. Next, grids were rinsed with blocking solution. As an optional step to demonstrate the presence of primary antibody coating the grid surface, some grids were incubated on droplets of 10 nm gold‐conjugated rabbit anti‐goat IgG (Electron Microscopy Sciences) diluted 1:20 in blocking solution for 20 min, and then rinsed with blocking solution (Figure S3C). Grids were then negative stained as describe above. As a control for the specificity of KIT‐EV binding to antibody‐coated grids, primary antibody was omitted during the antibody coating step (PBS only) prior to blocking with BSA and incubation with EV fractions and 10 nm gold‐conjugated secondary antibody. On control grids, vesicles or immunogold particles were very rarely observed (see Figure S3B,C). Grids were examined using a ThermoFisher Tecnai T20 transmission electron microscope operated at 200 KV, and images collected using a NanoSprint12 CMOS TEM camera (Advanced Microscopy Techniques). Images were adjusted for display using Photoshop 2021 (Adobe). Vesicle size distributions were analyzed using Fiji image analysis software.

### 2.13 Statistical analysis

Statistical analysis was completed with GraphPad Prism software (version 8.4.2) using an unpaired, two‐tailed Student's t‐test. All experiments were performed at least at three independent times.

### 2.14 Supporting information

We have submitted all relevant data of our experiments to the EV‐TRACK knowledgebase (EV‐TRACK ID: EV220187) (Van Deun et al., 2017).

The mass spectrometry proteomics data have been deposited to the ProteomeXchange Consortium via the PRIDE (Perez‐Riverol et al., 2022) partner repository with the dataset identifier PXD033889 and PXD033890.

## 3 RESULTS

### 3.1 EVs secreted by neoplastic huMCs lines contain KIT and canonical EV markers

**FIGURE 1:**
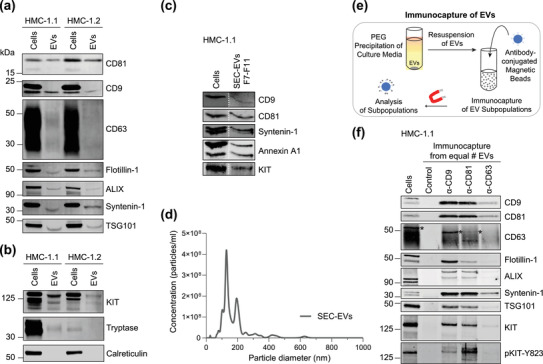
HMC‐1‐derived EVs contain KIT and canonical EV protein markers. (A, B) Detection of canonical EV marker (A) and mast cell marker proteins (B) as well as calreticulin as an exclusion marker in HMC‐1.1 and HMC‐1.2 cells and secreted EVs. 30 μg of protein of cell or EV lysates were separated by SDS‐PAGE, blotted and probed with the indicated antibodies. (C) HMC‐1.1‐derived EVs were separated from soluble protein by size exclusion chromatography (SEC; qEVoriginal70) and eluted as pooled fractions F7‐F11, followed by filter concentration. Cell (30 μg) and EV lysates (15 μg) were analyzed by immunoblotting with the indicated antibodies. The dashed line indicates the samples were run on the same blot, but not in contiguous lanes. (D) A representative graph showing the concentration and size measurement of SEC‐isolated EVs (C) by nanoparticle tracking. The average profile of five workflow repetitions is displayed. (E) Workflow of the immunocapture of EV subpopulations. EVs are bulk‐isolated from pre‐cleared cell culture media by PEG‐precipitation and incubated with antibody‐conjugated beads allowing for immunocapture of specific EV subpopulations. (F) Representative Western blot of CD9‐, CD81‐ and CD63‐immunocaptured EVs. Each bead capture was performed from an equal number of HMC‐1.1‐derived EVs (1.9 × 10^10^ EVs). Unconjugated beads served as a negative control. The presence of canonical EV protein markers as well as KIT in the EV subpopulations were determined by immunoblotting. *, signal from previous probing with a flotillin‐1 antibody

Neoplastic huMCs have been reported to release high numbers of EVs. To confirm the presence of classical EV protein markers, we initially used polymer‐assisted precipitation to isolate EVs secreted into the cell culture media by the neoplastic huMC lines HMC‐1.1 and HMC‐1.2. In agreement with earlier studies on the parental HMC‐1 cell line (Lázaro‐Ibáñez et al., 2019; Shelke et al., 2019; Xiao et al., 2014), immunoblotting revealed that EVs derived from the two sublines contained canonical EV protein markers, such as the CD81, CD9, and CD63 tetraspanins (Figure 1A). The membrane protein flotillin‐1 and other EV biogenesis factors like ALIX, syntenin‐1 and TSG101 were also present in EV preparations (Figure 1A), while calreticulin, an endoplasmic reticulum protein used as a negative EV marker, was absent (Figure 1B). We also confirmed that typical mast cell proteins such as KIT and tryptase were associated with HMC‐1.1 and HMC‐1.2 EVs (Figure 1B). However, the expression levels of KIT and tryptase were higher in HMC‐1.1 cell lysates and EVs (Figure 1B). Based on this observation, we subsequently focused on the HMC‐1.1 cell line.

We further verified that KIT was present together with canonical EV markers in HMC‐1.1‐derived EVs isolated by SEC (Figure 1C), suggesting that KIT is a *bona* *fide* protein contained in HMC‐1.1 EVs, which ranged in size from 100 to 200 nm as determined by nanoparticle tracking (Figure 1D).

Immunoaffinity capture of EVs using generic or cell type‐specific surface markers has become an approach of choice for isolating specific and pure EV subpopulations. To isolate EV subsets by immunocapturing, we first precipitated EVs secreted by HMC‐1.1 cells with 8% polyethylene glycol (PEG) to yield a high recovery of EVs (Figure 1E). In line with other reports (Rider et al., 2016; X. Zhang et al., 2020), this approach proved to be more economical and efficient for the isolation of total EVs than a commercial EV isolation reagent, as indicated by the increased abundance of common EV protein markers (Figure S1A). Nanoparticle tracking of EVs enriched by 8% PEG showed a peak size of about 200 nm (Figure S1B). We then combined the enriched EVs with magnetic beads conjugated to CD9, CD63 or CD81 antibodies to capture EV subpopulations that possess these membrane protein EV markers (Figure 1E). Western blot analysis showed that CD9‐ and CD81‐positive EVs were successfully isolated and contained similar quantities of canonical EV protein markers except for flotillin‐1, which was less abundant in CD81‐captured EVs (Figure 1F). Of note, CD9‐, CD81‐, and CD63‐immunopurified EV populations harboured the membrane‐embedded receptor KIT, which was also found in its active phosphorylated form (Figure 1F). The isolation of CD63‐positive vesicles was less efficient, which could be due to the quality of the conjugated antibody, the topology and/or accessibility of CD63 within the vesicle membrane. These results confirm that vesicles released by neoplastic HMC‐1.1 cells contain KIT and other canonical EV markers and present a proof‐of‐concept that mast cell‐derived EV subsets can be isolated by immunocapture.

### 3.2 Immunocapture enables the isolation of KIT‐containing EV subpopulations

**FIGURE 2:**
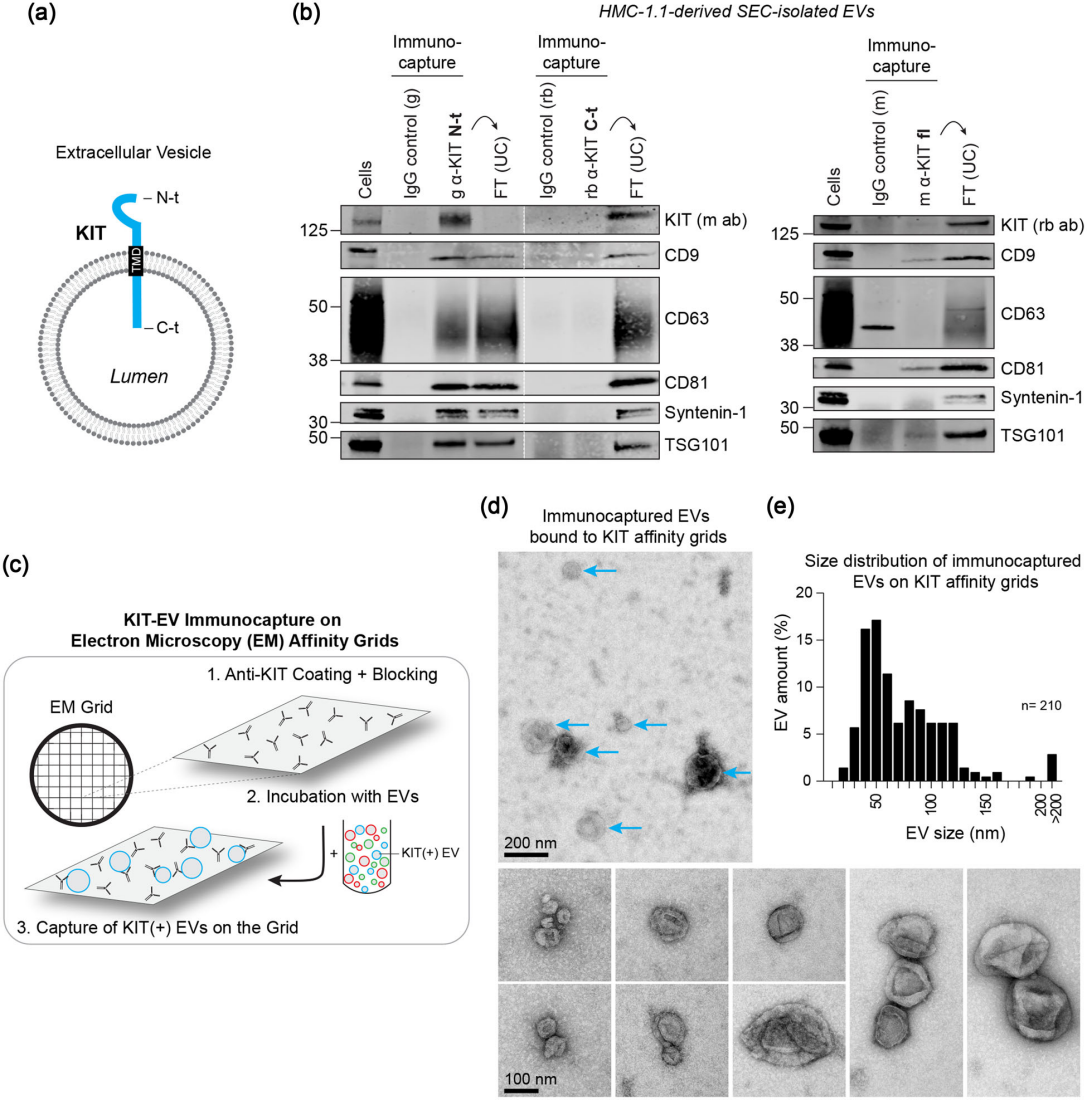
KIT‐containing EVs can be isolated by immunocapture. (A) Illustration of the predicted topology of the receptor KIT in an EV. KIT is embedded in the EV membrane by its transmembrane domain (TMD) with the N‐terminal (N‐t) ligand‐binding domain exposed to the extravesicular environment, while the C‐terminal (C‐t) protein domain is directed into the EV lumen. (B) Representative immunoblots showing the canonical EV marker content of KIT‐EVs captured from HMC‐1.1‐derived EVs that were isolated by size exclusion chromatography (SEC). Three different antibodies directed against N‐t, C‐t (left panel) or full‐length (fl) KIT protein (right panel) were used for immunocapturing of KIT‐containing EVs. Each pulldown was performed from vesicles secreted by 20 × 10^6^ cells and collected in SEC fractions seven to nine. Unbound EVs in the flow‐through (FT) were pelleted by ultracentrifugation (UC) to demonstrate the capture efficiency. Beads conjugated to isotype control antibodies served as negative controls. Cell lysates (30 μg) were included. g, goat; rb, rabbit; m, mouse. Dashed lines imply lanes from the same blot that were not loaded contiguously. (C) Schematic of the capture of KIT‐EVs by electron microscopy (EM) KIT immunocapture grids. EM grids were coated with a primary KIT antibody (targeting the N‐terminus of KIT) followed by BSA‐blocking and then incubation with PEG‐precipitated EV preparations. Captured KIT‐EVs were visualized by negative stain EM. (D) Representative images of HMC‐1.1‐released vesicles immunocaptured on KIT affinity grids and visualized by negative stain EM. A field of view containing several KIT‐EVs (blue arrows) is shown above, and a montage of KIT‐EVs below. Scale bars are indicated. (E) Size distribution of immunocaptured EVs on KIT affinity grids presented as percentage. A total of 210 EVs (single and grouped vesicles) was counted

HuMC‐derived EVs can transfer KIT to other cell types altering the recipient cell phenotypes and potentially contributing to the pathogenesis of mastocytosis (Kim et al., 2018; Kim et al., 2021; Xiao et al., 2014). Enrichment of KIT‐containing exosomes from GIST cell lines and patient plasma has also been reported (Atay et al., 2018). Therefore, we asked whether KIT antibodies could be used for isolating and characterizing huMC‐derived KIT‐containing vesicles (Figure 2A). In concordance with MISEV2018 guidelines (Théry et al., 2018), we used antibodies directed against the N‐terminus, C‐terminus, or the full‐length KIT protein, an approach that would confirm the predicted topology of KIT within vesicles and has not been previously addressed (Atay et al., 2018). As expected, the antibody recognizing the N‐terminal ligand‐binding domain of KIT, facing the extravesicular milieu, co‐precipitated the largest quantity of EV proteins from SEC‐isolated or PEG‐precipitated EVs (Figure 2B, left and Figure S2A). Sedimentation of unbound EVs in the flow‐through by ultracentrifugation demonstrated that the N‐terminal KIT capturing antibody efficiently depleted KIT‐EVs (Figure 2B). On the contrary, the antibody recognizing the C‐terminus of KIT, predicted to be intravesicular, did not immunoprecipitate KIT or EV markers from intact vesicles, but these proteins were sedimented from the flow‐through EVs (Figure 2B, left and Figure S2A,B). Only when C‐terminal epitopes became accessible by lysing EVs with Triton X‐100 prior to the immunocapture, the C‐terminal KIT antibody was able to precipitate KIT from EV preparations (Figure S2B). Similarly, immunocapture with a KIT antibody recognizing full‐length protein precipitated small quantities of KIT and other protein markers, which was expected if a part of the receptor is intravesicular and inaccessible to the antibody (Figure 2B, right). These results support the conclusion that the N‐terminal domain of KIT is mostly oriented towards the extravesicular side. Like the CD9‐ and CD81‐immunocaptured EVs (Figure 1F), the isolated KIT‐vesicles contained canonical EV tetraspanins and other EV markers such as flotillin‐1, syntenin‐1 and TSG101 (Figures 2B and S2A), suggesting an overlap in content between these populations. These results highlight that immunocapture can selectively and efficiently isolate KIT‐positive EV subpopulations secreted by neoplastic huMCs.

The morphology of HMC‐1.1‐derived KIT‐EVs was examined by negative stain EM. KIT‐EVs were immunocaptured on antibody‐coated affinity grids (Derrick, 1973; Yu et al., 2016), prepared by coating EM grids with a KIT antibody followed by BSA blocking (Figures 2C and S3). BSA blocking prevented non‐specific binding of KIT‐EVs to grids that were not coated with the KIT antibody (Figure S3B,C, Control), but it did not impede specific binding of KIT‐EVs to KIT immunocapture grids (Figures 2D and S3B,C). The EVs immunocaptured on the KIT affinity grids displayed varying vesicle sizes and did not show major morphological variations (Figure 2D). In general, they appeared as cup‐shaped, round to oval dehydrated vesicles with darker staining around the membranes as expected in negative stain EM (Figure 2D). While the majority of KIT‐EVs were 30–120 nm in size, we also found vesicles that were larger than 200 nm (Figure 2E). We also noticed that the vesicles were commonly detected in groups, suggesting that some EVs were retained on the grid due to interaction between vesicles.

Together, our experiments provide evidence that antibodies against the N‐terminal domain of the KIT protein can be used to selectively capture KIT‐EV populations by immunoaffinity beads or EM affinity grids.

### 3.3 KIT‐captured EVs reveal a distinct EV marker profile

**FIGURE 3:**
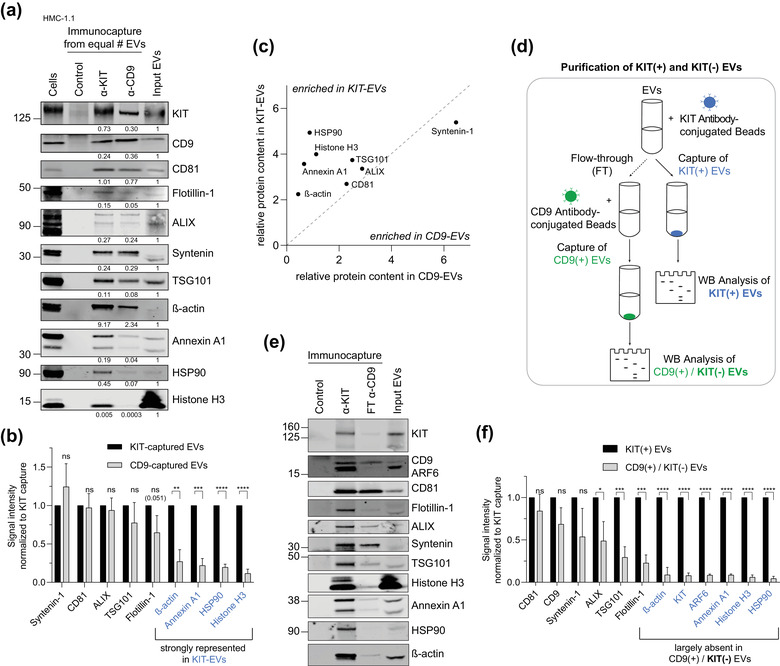
KIT‐captured EVs reveal distinct EV marker profiles compared to CD9‐enriched vesicles. (A) Comparison of KIT‐ vs. CD9‐captured EV populations. Each EV immunoprecipitation was performed from an equal number of EVs (3.5 × 10^10^ EVs), and the protein content was assessed by immunoblotting. Unconjugated beads served as a negative control. KIT‐EV immunocapture was performed with an antibody against N‐terminal KIT. Representative blots are shown. Signal intensities of precipitated proteins were normalized to the adjusted shown input (the input equalled 8.9% of the starting material of an IP) and indicated below each blot. Cell lysates (30 μg) were loaded in the first lane. (B) Quantification of protein signal intensities (A) in CD9‐EVs compared to KIT‐EVs. Data are represented as mean ± S.D. from three independent experiments. Proteins that are strongly represented in KIT‐EVs and reduced in CD9‐captured EVs are highlighted in blue. (C) Correlation plot representing the relative enrichment of probed proteins in KIT‐ vs. CD9‐EVs. (D) Illustration of the isolation procedure of KIT‐positive (KIT(+)) and KIT‐depleted (KIT(‐)) EVs. EVs were first immunocaptured by KIT‐conjugated beads and total eluates were analyzed. The flow‐through, containing KIT‐depleted EVs, was further processed by immunocapture with CD9‐conjugated beads yielding CD9‐positive/KIT‐negative (CD9(+)/KIT(‐)) EVs. (E) Representative immunoblot of bead‐captured KIT‐positive (second lane) and CD9(+)/KIT(‐) EVs from the flow‐through (third lane). Unconjugated beads were used as a negative control (first lane). KIT immunocapture was performed with an antibody against N‐terminal KIT from 2.4 × 10^10^ EVs. FT, flow‐through. (F) Signal intensities of protein markers (E) were quantified and are represented as mean ± S.D. from three independent experiments and normalized to KIT(+) EVs. Protein markers largely absent from KIT‐depleted EVs are highlighted in blue. Ns, not significant; *p < 0.05; **p <0.01; ***p < 0.001; ****p < 0.0001 (unpaired Student *t*‐test)

To determine if the protein content of KIT‐containing vesicles differs from other EV subpopulations secreted by HMC‐1.1 cells, we analyzed several additional protein markers in KIT‐ and CD9‐immunopurified vesicles. Both KIT‐ and CD9‐captured vesicle populations contained comparable amounts of the putative exosome markers CD81, ALIX, syntenin‐1, and TSG101 (Figure 3A‐C). However, KIT‐EVs were notably enriched in β‐actin, annexin A1 and heat‐shock protein 90 (HSP90), which are suggested not to be associated with classical (low‐density) exosomes (Jeppesen et al., 2019), and histone H3 (Figure 3A‐C). Although there are no consensus protein markers yet that explicitly discriminate between exosomes and microvesicles, the presence of putative exosomal and non‐exosomal proteins indicated that the immunocaptured KIT‐EVs are likely heterogeneous and consist of EVs derived from endocytic/MVB and non‐endocytic origin.

The KIT‐ and CD9‐captured EVs both contained CD9 as well as KIT (Figure 3A‐C), suggesting they may overlap to some extent. To investigate the potential overlap or difference between these subpopulations, we used the immunocapture method to isolate first KIT‐EVs from the HMC‐1.1‐derived vesicles and then performed CD9 immunocapturing on the flow‐through to isolate KIT‐depleted/CD9‐positive EVs (Figure 3D). This approach revealed that histone H3, annexin A1, HSP90 and β‐actin were almost exclusive to KIT‐containing vesicles (Figure 3E,F), whereas CD81, CD9 and other canonical EV markers were present in both KIT‐positive and KIT‐negative/CD9‐positive EVs (Figure 3E,F). We also found that KIT‐positive EVs contain the small GTPase ADP‐ribosylation factor 6 (ARF6) (Figure 3E,F), which has been implicated in the shedding of (tumour‐derived) microvesicles (Clancy et al., 2019; Muralidharan‐Chari et al., 2009). The overall absence of histone H3, annexin A1, HSP90, β‐actin and ARF6 in KIT‐depleted/CD9‐positive EVs may suggest this subpopulation is mostly of exosomal origin. While the relatively low abundance of these proteins in KIT‐negative/CD9‐positive EVs captured from the flow‐through could be due to a partial pre‐depletion of EVs (Figure 3E), similar patterns of selective markers between KIT‐ and CD9‐EVs were observed when captured from equal EV numbers (Figure 3A,B), supporting distinct differences between these populations and also highlighting the heterogeneity of CD9‐containing EVs. The protein content in KIT‐EVs was confirmed using proteomic analysis (Figure S4A,B and S5). Moreover, we obtained similar data when comparing KIT‐positive and KIT‐negative/CD81‐positive EVs (Figure S4C). These results suggest that heterogeneous KIT‐containing EVs secreted by neoplastic huMCs are distinguished from KIT‐depleted EV populations by a microvesicle‐like protein profile.

### 3.4 KIT is incorporated into microvesicle‐ and exosome‐like subpopulations that possess different physical properties

**FIGURE 4:**
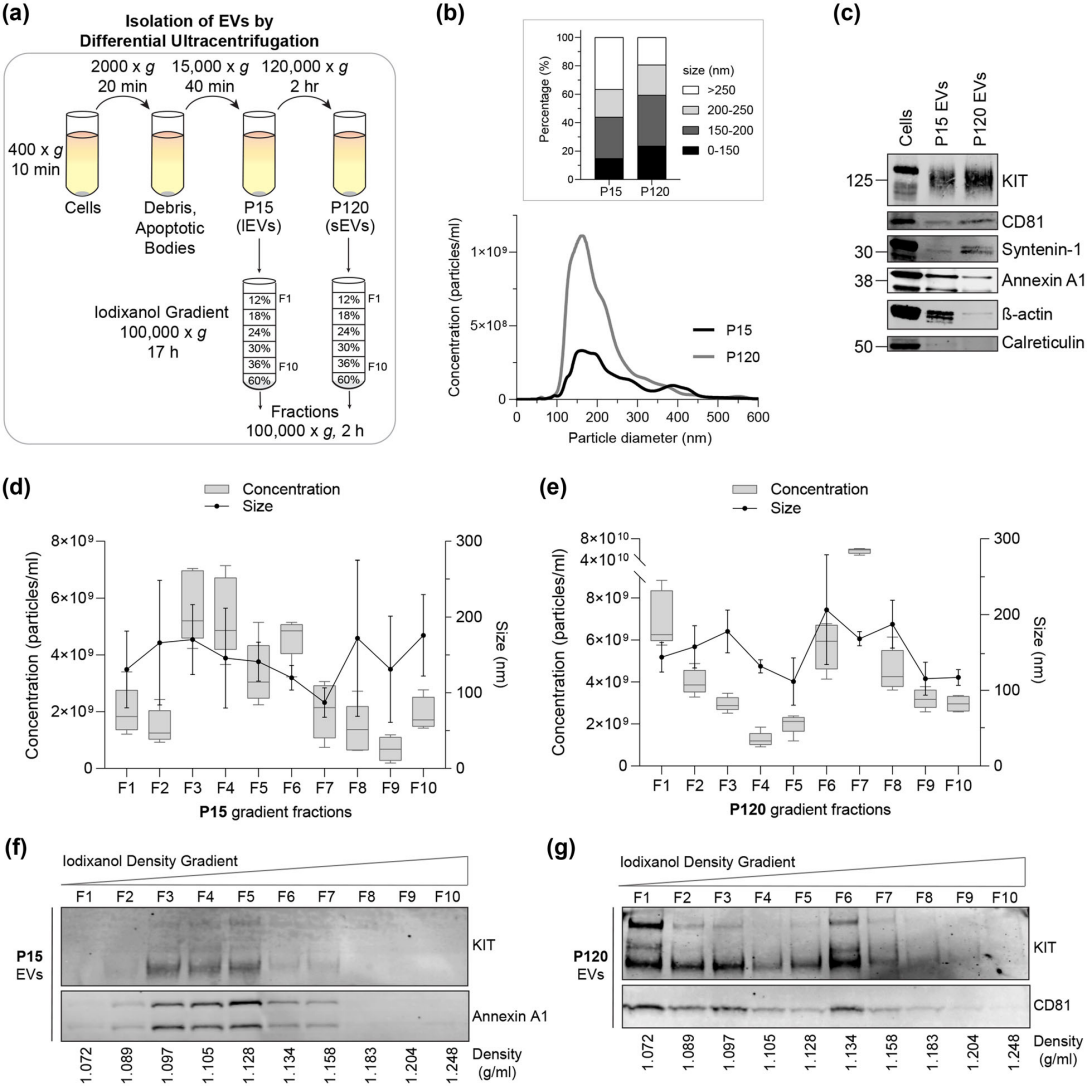
KIT‐EVs are released as heterogeneous subpopulations with different physical properties. (A) Cell culture supernatant was subjected to differential ultracentrifugation to obtain P15 and P120 EV pellets, as illustrated. Where indicated, P15 and P120 EVs were further separated by iodixanol density gradients. Vesicles from each density fraction were pelleted and analyzed. (B) P15 and P120 EVs obtained from HMC‐1.1 cells were analyzed by nanoparticle tracking. Representative concentration and size profiles averaged from five workflow repetitions are depicted. The averaged percentage (%) of different size ranges within the P15 or P120 population is shown on top (bar chart). (C) Representative Western blot of HMC‐1.1 cell, P15 and P120 EV lysates (30 μg) probed with the indicated antibodies. (D, E) HMC‐1.1‐derived P15 (D) or P120 (E) EV pellets were separated by density gradients and the vesicles from each fraction (F1‐F10) were analyzed by nanoparticle tracking for concentration (particles/ml), shown as box plots, and size (represented as average mode, nm), shown as a line graph. Data in the box plots represent the median with the whiskers indicating minimum and maximum values; data in the line graph are represented as mean ± S.D. from five repetitions. Representative results are shown. (F, G) Representative immunoblots showing the presence of KIT and annexin A1 in P15 EV fractions (F) and KIT and CD81 in P120 EV fractions (G). The density (g/ml) of each fraction is indicated below

Based on the observation that KIT‐EVs bear characteristic markers of both exosome‐ and microvesicle‐like EVs, we employed differential ultracentrifugation to separate these subtypes. To enrich microvesicle‐like EVs (“large” EVs), pre‐cleared HMC‐1.1 cell media was sedimented at 15,000 × *g* (P15 EVs) and exosome‐like EVs (“small” EVs) were enriched by pelleting the P15 supernatant at 120,000 × *g* (P120 EVs) (Jeppesen et al., 2019; Kowal et al., 2016) (Figure 4A, top). Nanoparticle tracking of the EV groups revealed that 40% of P15 EVs and 60% of P120 EVs were smaller than 200 nm and that the P120 fraction possesses a higher vesicle concentration (Figure 4B). As expected, P120 EVs showed an enrichment for classical exosome markers (such as syntenin‐1), whereas P15 EVs showed a bias for putative microvesicle markers (β‐actin and annexin A1), and neither EV group contained the negative marker calreticulin (Figure 4C). Importantly, KIT was present in both the P15 and P120 EVs (Figure 4C), indicating that KIT exists in both exosome‐ and microvesicle‐like EVs.

To further investigate the physical properties of KIT‐containing P15 and P120 EVs, we applied density gradient centrifugation on both EV pellets (Figure 4A, bottom). The concentration of separated P15 EVs peaked between fractions of three and six with an average size range of 150–180 nm (Figure 4D). The highest levels of KIT‐ and annexin A1 were also found in these lower density (1.09–1.12 g/ml) fractions, supporting that KIT‐EVs in the P15 group are likely microvesicles (Figure 4F). P120 vesicles containing KIT were found in lower density fractions (1.07–1.09 g/ml) with an average size of 150 nm, and also peaked in the higher density (1.13 g/ml) fraction six where the vesicles possessed an average size of 200 nm (Figure 4E,G). CD81 coincided with the KIT‐containing P120 EV fractions suggesting that the KIT‐EVs in the P120 group are of exosomal origin (Figure 4G).

**FIGURE 5:**
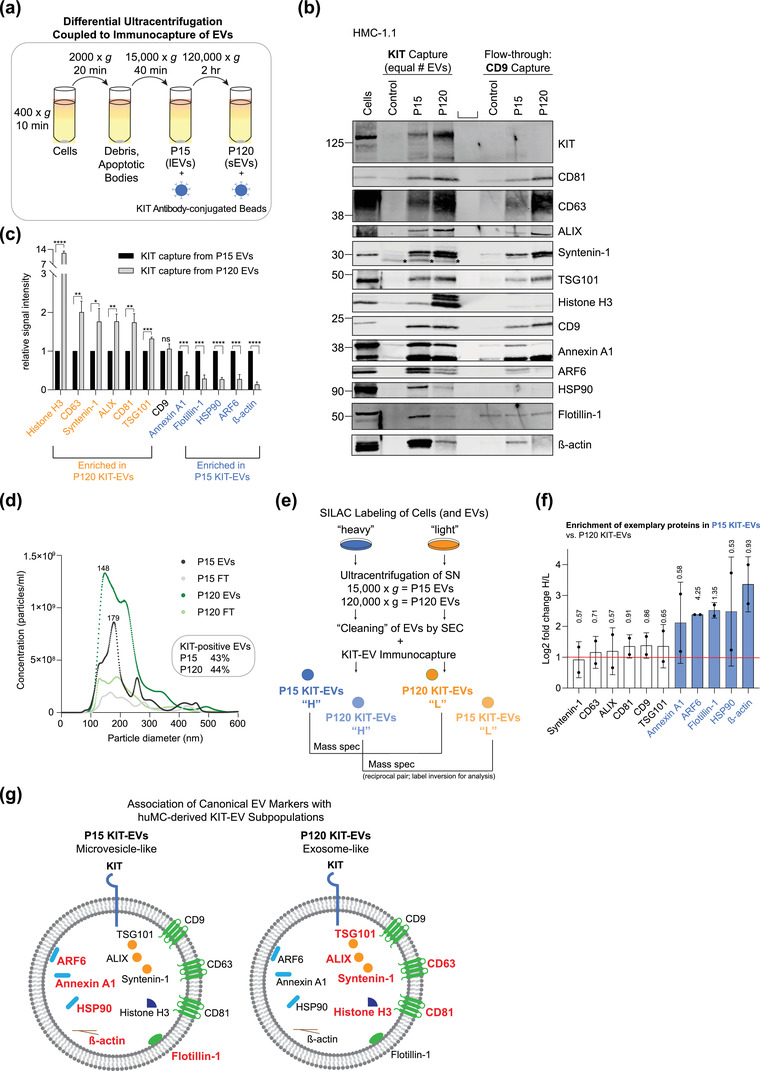
Mast cells produce microvesicle (P15)‐ and exosome (P120)‐like KIT‐EVs. (A) Schematic of KIT immunoprecipitation from P15 and P120 EV populations. (B) KIT‐EVs were purified from P15 or P120 EVs (8.9 × 10^9^ EVs) by immunoprecipitation with an antibody against N‐terminal KIT. EVs in the flow‐through were captured with CD9‐conjugated beads. Unconjugated beads and a mix of P15 and P120 vesicles were used as a negative control. Cell lysates (30 μg) and EV eluates were analyzed by Western blotting with the indicated antibodies. Representative blots are shown. *, indicates bands arising from protein G. (C) Relative signal intensities of protein markers shown in (B) were quantified and normalized to signals in P15 KIT‐EVs. Data are shown as mean ± S.D. from three independent experiments. Proteins enriched in P120 and P15 KIT‐EVs are highlighted in orange and blue, respectively. Ns, not significant; *p < 0.05; **p < 0.01; ***p < 0.001; ****p < 0.0001 (unpaired Student *t*‐test). (D) The concentration of P15 and P120 EVs before and after KIT immunoprecipitation shown in (B) was measured by nanoparticle tracking. The representative profile shows the distribution of particle numbers for a range of particle sizes averaged from five workflow repetitions. The sizes (nm) of the most abundant particles in P15 and P120 are indicated. The percentage of KIT‐EVs was estimated by determining the particle count before and after KIT‐EV capturing. FT, flow through. (E) After SILAC labelling of HMC‐1.1 cells with heavy and light amino acids, EVs were isolated from the cell culture supernatant (SN) by ultracentrifugation as P15 and P120 EV pellets. EV suspensions were further cleaned by size exclusion chromatography (SEC) before performing KIT immunocapturing on each EV pool. Heavy‐labeled KIT P15 EVs were mixed with light‐labelled KIT P120 EVs (and vice *versa*) and processed for quantitative mass spectrometry as inverse duplicates. (F) The enrichment of exemplary proteins (presented as log2 fold change H/L) determined by SILAC mass spectrometry in P15 KIT‐EVs compared to P120 KIT‐EVs is shown. Data are presented as the mean from two mass spectrometry pairs ± S.D. The red line indicates the 2‐fold enrichment threshold. Proteins highly enriched in P15 KIT‐EVs are indicated in blue. The ‐Log T‐test p‐value is shown above each bar. ‐Log T‐test p‐values > 1.3 indicate significance. (G) Illustration of P15 and P120 KIT‐EVs summarizing the association of EV markers with respective vesicle types. Protein markers highly associated with either P15 (microvesicle‐like) or P120 (exosome‐like) EVs are highlighted in red

To examine the composition of KIT‐positive P15 and P120 EVs, we immunocaptured KIT‐EVs from both EV groups and probed for several protein markers (Figure 5A). The immunocapture efficiently isolated the KIT‐EVs from the P15 and P120 groups as KIT was not detectable in the CD9‐captured vesicles from the flow‐through (Figure 5B; also see Figure 2B). In the P15 KIT‐EVs we observed an enrichment for markers generally associated with microvesicles, including annexin A1, ARF6, HSP90, and β‐actin (Figure 5B,C). In contrast, the P120 KIT‐EVs showed a higher representation of classical MVB/exosome markers such as CD81, CD63, ALIX, syntenin‐1 and TSG101 (Figure 5B,C). Notably, nanoparticle tracking of the EVs before and after the KIT immunocapture suggested that KIT‐EVs are abundant and represent approximately 40% of the HMC‐1.1‐secreted EVs (Figure 5D).

We also performed quantitative mass spectrometry comparing P15 and P120 KIT‐EVs isolated from the cell culture media of SILAC‐labelled HMC‐1.1 cells (Figure 5E). Our proteomic analysis confirmed that the P15 KIT‐EVs were enriched for annexin A1, ARF6, flotillin‐1, HSP90 and beta‐actin (Figure 5F). Further analysis using normalized H/L ratios indicated that the most upregulated GO term for the P120 KIT‐EV fraction was “ESCRTI complex”, while the P15 KIT‐EV fraction was associated with the terms “actin filament and cytoskeleton” (Figure S5C,D).

Together, these data support the conclusion that neoplastic huMCs release in vitro an abundant pool of heterogeneous KIT‐EVs consisting of both microvesicle (P15)‐ and exosome (P120)‐like EV subpopulations (Figure 5G) that potentially perform different biological functions.

### 3.5 Inhibition of neutral and acid sphingomyelinase affects the protein loading of KIT‐EV subtypes

**FIGURE 6:**
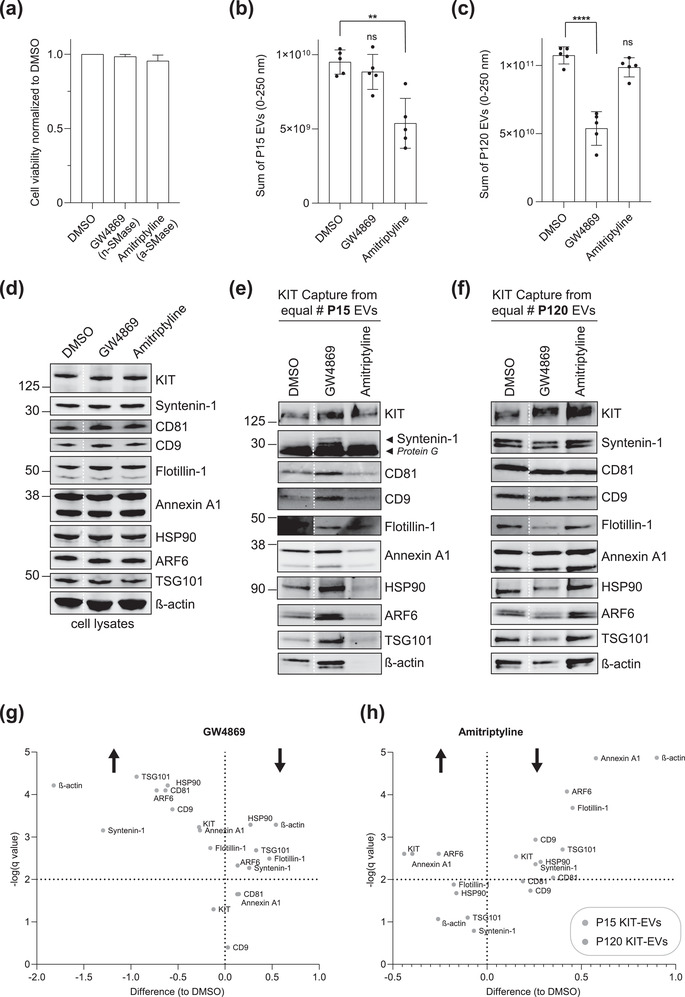
The protein loading of KIT‐EVs shifts among vesicle subtypes after cell treatment with SMase inhibitors. (A) Viability assessment of HMC‐1.1 cells at the end of the 16 h treatment with DMSO (vehicle control), 10 μM GW4869 (n‐SMase inhibitor) or 10 μM amitriptyline (a‐SMase inhibitor) to control for potential drug‐induced cell death. Data are presented as mean ± S.D. from three independent experiments, normalized to the control. (B, C) EVs released by control‐ or inhibitor‐treated HMC‐1.1 cells were pelleted as P15 (B) or P120 (C) EVs and evaluated by nanoparticle tracking. The sum of P15 (B) or P120 (C) EVs (ranging from 0 to 250 nm) released under each condition was plotted. Representative data are shown as mean ± S.D. from five repetitions. Ns, not significant; **, 0.0011; ****p < 0.0001 (two‐tailed, unpaired Student *t*‐test). (D) Total lysates (30 μg) of DMSO‐ or inhibitor‐treated cells were analyzed by immunoblotting with the indicated antibodies. (E,F) KIT‐EVs were captured with an antibody against N‐terminal KIT from P15 (3.4 × 10^8^ EVs) (E) or P120 (2.6 × 10^9^ EVs) (F) vesicles released by control, GW4869‐ or amitriptyline‐treated cells. Total KIT‐EV lysates were evaluated by Western blotting; representative blots are shown. (G,H) Signal intensities of proteins in KIT‐EVs (E,F) were quantified and normalized to the DMSO control. Up‐ or down‐regulation (indicated by arrows) of protein content in P15 and P120 KIT‐EVs in response to GW4869 (G) or amitriptyline (H) is displayed in Volcano graphs (*n* = 3). The *q*‐value is the FDR‐adjusted *p*‐value, and the dashed line indicates the significance threshold. In D, E and F, dashed lines imply lanes from the same blot that were not loaded contiguously

Inhibition of neutral (n)‐sphingomyelinase (SMase) by GW4869 reduces the generation of MVB‐derived exosomes, whereas the inhibition of acid (a)‐SMase by amitriptyline impairs the production of microvesicles at the plasma membrane (Catalano & O'Driscoll, 2020; Verderio et al., 2018). Therefore, to gain further insight into the biogenesis of KIT‐EVs and to determine whether KIT can shift between EV subtypes, we tested the effect of SMase inhibitors on the protein content of KIT‐EVs. Treatment with GW4869 or amitriptyline did not markedly influence the viability of HMC‐1.1 cells (Figure 6A). However, nanoparticle tracking of the P15 and P120 EVs showed that, as expected, amitriptyline significantly reduced the number of P15 (microvesicle‐like) EVs (Figure 6B), while GW4869 substantially decreased the quantity of released P120 (exosome‐like) vesicles by 50% (Figure 6C). Based on the observed selective effects of these SMase inhibitors (Figure 6B,C) and their known mechanism of action, these data further support the conclusion that P15 and P120 EVs released by HMC‐1.1 cells largely represent microvesicle‐ and exosome‐like vesicles, respectively.

We then investigated whether KIT and other EV content would shift between EV subtypes when using these inhibitors. While neither inhibitor altered the protein expression of vesicle markers or KIT in total cell lysates (Figure 6D), the relative composition of KIT‐EVs immunocaptured from P15 or P120 EVs changed (Figure 6E‐H). Amitriptyline significantly reduced KIT and several EV protein markers in P15 KIT‐positive EVs, but these proteins tended to increase in the P120 KIT‐positive EVs (Figure 6E,F,H). Conversely, GW4869 significantly increased KIT and other protein markers in P15 KIT‐EVs (Figure 6E,G), whereas P120 KIT‐EVs contained, by trend, reduced levels of cargo protein (Figure 6F,G). These observations suggest that perturbing the biogenesis pathways of either microvesicles or exosomes by SMase inhibitors results in a shift of the protein cargo between KIT‐EV subtypes as has been observed for EVs in other cell types (Catalano & O'Driscoll, 2020; Menck et al., 2017), proposing the presence of regulatory processes that link microvesicle and exosome secretion. However, these treatments did not prevent the release of KIT in a particular EV subtype. These results do not exclude that KIT‐EVs may also be formed by lipid‐independent pathways since membrane budding at the MVB or plasma membrane can also be facilitated by the ESCRT machinery (Mathieu et al., 2019).

### 3.6 Proteomic profiling of KIT‐EVs

**FIGURE 7:**
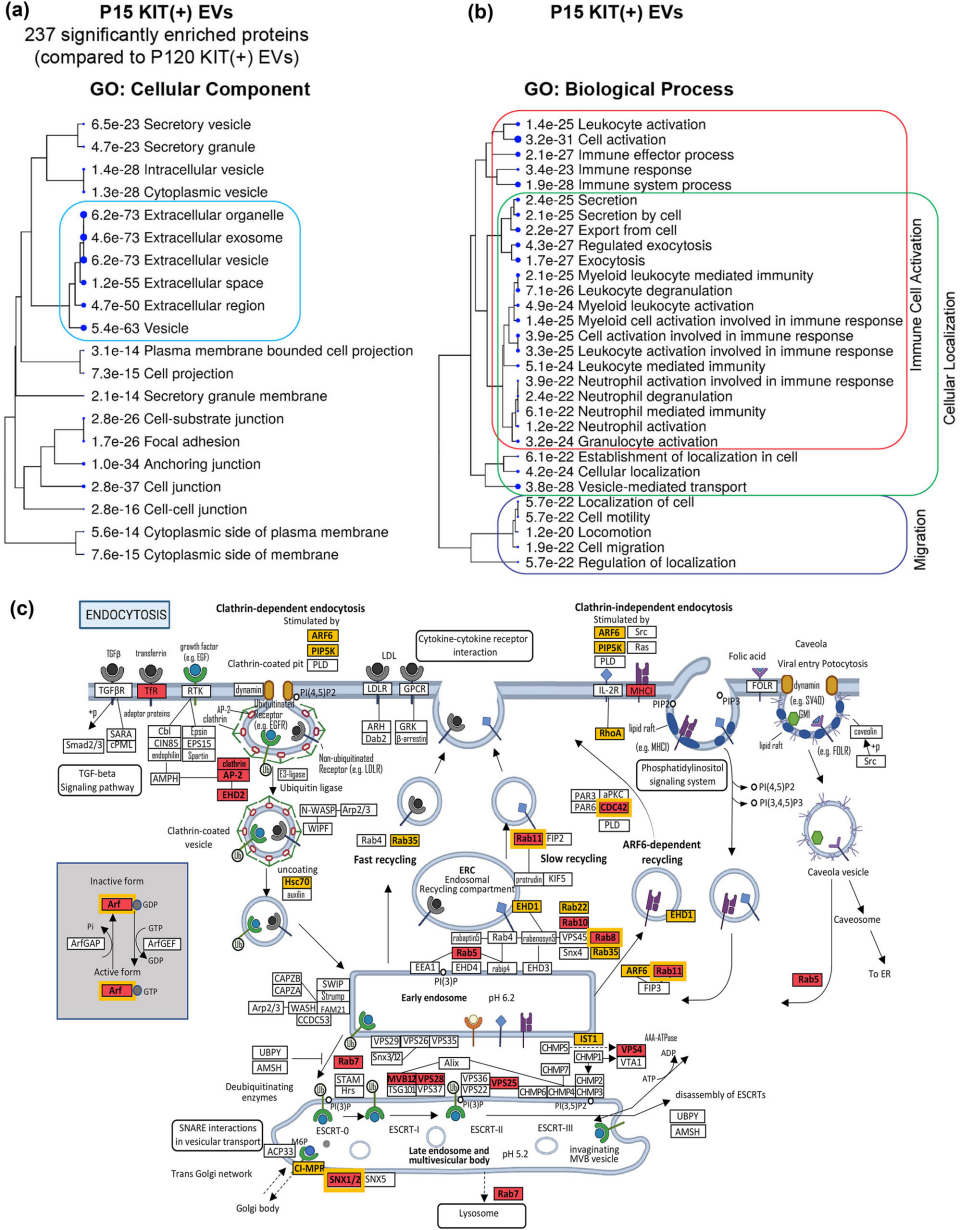
Proteomic profiling of P15 KIT‐EVs. (A, B) Hierarchical clustering trees summarizing the correlation among significant pathways for the indicated GO‐terms. Pathways with shared genes are clustered. Larger dots indicate higher significance. (A) Hierarchical clustering tree of GO “cellular component” terms of significantly enriched proteins in P15 KIT‐EVs (compared to P120 KIT‐EVs). (B) Hierarchical clustering tree of GO “biological process” terms of significantly enriched proteins in P15 KIT‐EV. Larger clusters are highlighted by boxes. (C) A KEGG pathway analysis was performed for enriched proteins in KIT‐EVs (in comparison to KIT‐depleted EVs) and in P15 KIT‐EVs (in comparison to P120 KIT‐EVs). The KEGG pathway “endocytosis” (map 04144) is shown. This pathway was modified using Biorender.com (agreement #MY23SQ80ZR) and Microsoft PowerPoint (v16.60). Enriched proteins in KIT‐EVs found in this pathway are highlighted in red, those enriched in P15 KIT‐EVs compared to P120 KIT‐EVs are highlighted in yellow. Boxes in red with a double yellow border indicate that the protein is enriched in both populations

Proteomic analysis confirmed the presence of characteristic markers of microvesicles and exosomes in KIT‐EVs (Figures 5F, S4B and S5C). In addition, gene ontology (GO) analysis performed with significantly enriched proteins in KIT‐EVs (compared to KIT‐depleted/CD9‐positive EVs) or in P15 KIT‐EVs (compared to P120 KIT‐EVs) revealed that the most significant hits for GO “cellular component” were EV, extracellular exosome and extracellular organelle, underlining that our immunocapturing approach is a valid strategy for isolating EVs (Figures 7A and S6A).

We further analyzed the protein composition of KIT‐EVs beyond canonical vesicle markers to gain insight into potential biological functions. Significantly enriched proteins in KIT‐EVs, particularly in P15 KIT‐EVs, were related to GO “biological process” terms such as immune cell activation, cellular localization (vesicular trafficking), and cell migration (Figures 7B, S6B and Table S1). Closer evaluation indicated that a distinct characteristic of the genes enriched in P15 KIT‐EVs, and to some extent in total KIT‐EVs, was the presence of cell membrane receptors, including KIT, cytokine receptors, integrins and cell adhesion receptors, along with numerous proteins involved in the signalling of those receptors, such as adaptor proteins, protein kinases, phosphatases, and G‐proteins (Table 1). The existence of cell membrane receptors and signalling components in immunocaptured KIT‐EVs and P15 KIT‐EVs suggests that their signature composition may also determine the ability to target specific cell types (via cell‐cell interaction receptors) and to execute distinct biological functions. The presence of receptors and signalling proteins was also described in the proteomic analysis of exosomes from GIST cell lines, which express mutant KIT (Atay et al., 2018). In comparison to GIST‐derived exosomes, huMC KIT‐EVs uniquely contain proteins distinct for mast cell functions, including ADGRE2 (EMR2), FCGR2, HPGD, L1CAM, SYK, BTK, CSK, PLCB4, LAT, LAT2, GRB2, TRPV2 and CACNA2D2 (Table S2). Conversely, from the list of proteins described as unique or novel to GIST exosomes (Atay et al., 2018), only KIT was commonly present in huMC KIT‐EVs, suggesting that different cell types expressing oncogenic KIT secrete EVs that represent the cell of origin in addition to common EV protein content (Table S2).

**TABLE 1:** Proteins within the indicated categories that are significantly enriched in KIT‐EVs compared to KIT(‐)/CD9(+) EVs; or in P15 KIT‐EVs compared to P120 KIT‐EVs

		**KIT‐EVs vs. KIT(‐)/CD9(+) EVs**				**P15 KIT‐EVs vs. P120 KIT‐EVs**			
**Protein type**	**Subtype**	**Enriched proteins**				**Enriched proteins**			
**Membrane** **receptors**	GPCRs	CD97				CD97	GPRC5C		
	Tyrosine Kinase Receptors	KIT				KIT	IGF2R	EPHB1	
	Immunoglobulin/ Cytokine Receptors	FCGR2A	FCGR2B			FCGR2A	FCGR2B	TNFRSF14	IL6ST
						IL10RB			
	Integrins/Extracellular Matrix Receptors	ITGA2	ITGA2B	ITGA4	ITGA5	ITGA2	ITGA2B	ITGA5	ITGAV
		ITGAV	ITGB1	ANTXR2	LAIR1	ITGB1	CD151	ANTXR2	LAIR1
	Cell‐Cell and Cell Adhesion Molecules	F11R	PLXND1	TPGB		F11R	CD99	PVRL2	JAM3
		CADM1	L1CAM			EPHB1	SIGLEC6	CADM1	ICAM
		CD81	CD82	CD59	CD97	CD44	CD97	CD82	CD59
		SIGLEC6	SIGLEC5 /14			PLXNB2	NECTIN2	SPN (CD43)	
**Transporters/Carriers/Channels**	ABC Transporters	ABCC4				ABCC1			
	Ion Channels	TRPV2	TMC8			TRPV2	TMC6	CLIC4	ANXA7
						ANXA5			
	Cation Transporter ATPases	ATP2B4	ATP1A1			ATP2B4	ATP2B1	ATP1A1	ATP1B3
	Solute/Molecule Transporters	SLC16A1/A3	SLC3A2	SLC1A4/ A5	SLC2A3/ A14	SLC16A1	SLC3A2	SLC7A5	SLC2A3
		SLC44A1				SLC2A14	SLC29A1	SLC12A7	IPO5
	Flippase					TMEM30A			
**Signalling proteins**	Protein Kinases	GRK6	PRKCB	PAK1	YES1	GRK6	PRKCA	FGR	YES1
		CSK	BTK	TNIK	TAOK3	LYN	CSK	SYK	JAK1
						TYK2	MAP4K4	NCK1	TAOK3
						TNIK	STK26	OXSR1	
	Protein Phosphatases	SIRPA				PTPRA	PPM1H	PTP4A1/A2	
	Lipid Kinases and Phospholipases	PLCB2	PLCB3			PIP5K1A	PI4KA	PIP4K2A/2B	
	Adaptor Proteins	AKAP2	PAG1	LAT	EFR3A	PAG1	LAT	LAT2	GRB2
						SH2D3C			
	G‐proteins Subunits and Regulators	GNB1	GNB2	GNAI2	GNAI3	GNB1	GNB2	GNAI2	GNAI3
		GNA13	GNAQ	GNAS		GNA11	GNA13	GNAS	GPSM3
	Transcription Factors					STAT3	STAT5A and B		
**Small G‐proteins and regulators**	Small G‐Proteins	ARF4	CDC42	RAB2A	RAB5C	ARF4	ARF6	CDC42	RAB6A/B
		RAB8A	RAB7A	RAB10	RAB11A/B	RAB8A/B	RAB11A/B	RAB13	RAB31
		RAB14	NRAS	KRAS	RALB	RAB35	NRAS	KRAS	RRAS
		RAP2B	RAP2C	SEPT11		RAP1A	RAP1B	RAP2A/2B	RHOA/C
						RHOG	SEPT2	SEPT9	SEPT11
	GDIs	GDI2	ARHGDIB						
	GEFs	DOCK11				SCRIB	DOCK10		
	GAPs	IQGAP1	IQGAP2	SRGAP2/2B/2C		RACGAP1	ARHGAP25	RANGAP1	RASAL3
						SRGAP2	ARHGAP18	RP2	

Abbreviations: ABC, ATP binding cassette family; GAP, GTPase activating protein, GDI, guanosine dissociation inhibitor; GEF, guanine nucleotide exchange factor.

Interestingly, a KEGG pathway analysis revealed that many of the significantly enriched proteins were related to the pathway “endocytosis,” giving insight into the origin of KIT‐EVs and the intracellular trafficking of KIT. Proteins enriched in KIT‐EVs are involved in the endocytosis of clathrin‐coated vesicles, recycling of endosomes, and in the formation of late endosomes and MVBs, whereas proteins enriched in P15 KIT‐EVs are mostly engaged in the recycling of endosomes (Figure 7C). These observations further support our conclusion that huMC KIT‐EVs encompass EVs originated from both MVBs/endosomes and the plasma membrane (i.e., exosomes and microvesicles, respectively), and that P15 KIT‐EVs largely represent microvesicles/ectosomes (derived from the plasma membrane).

### 3.7 KIT‐EV immunocapture from a cell model expressing normal KIT

**FIGURE 8:**
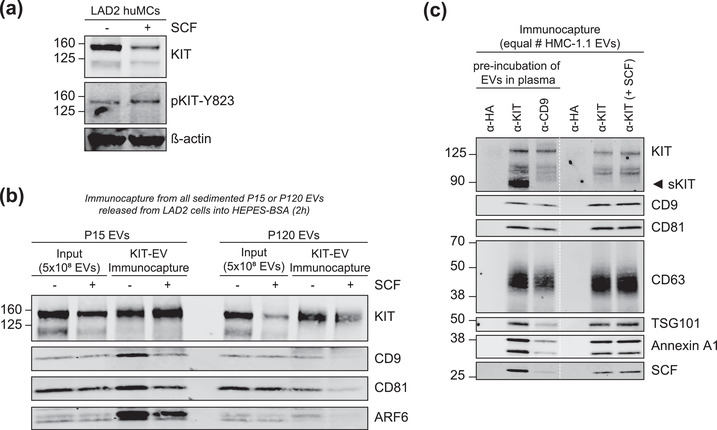
KIT‐EV immunocapture of LAD2 huMC EVs and KIT‐EV recovery from spiked plasma. (A) Cytokine‐starved LAD2 huMCs were treated or not with stem cell factor (SCF; 100 ng/ml) for 2 h in HEPES‐BSA buffer. Cell lysates (30 μg) were analyzed by immunoblotting with the indicated antibodies. (B) EVs released into the supernatant by 18 × 10^6^ LAD2 cells treated or not with SCF for 2 h were sedimented by differential ultracentrifugation into P15 and P120 EVs. KIT‐EV immunocapture was performed (KIT antibody AF332) from all sedimented LAD2 P15 and P120 EVs. The total bead elution was loaded and analyzed by Western blotting with the indicated antibodies. Input EV lysates (5 × 10^8^) were included. Representative blots are shown. (C) HMC‐1.1 cell line‐derived EVs (1.2 × 10^10^ EVs) were incubated or not in human normal plasma and captured with a KIT antibody (AF332) or by CD9 antibody‐conjugated beads. A goat HA antibody served as a negative isotype control. Representative blots are shown. sKIT, soluble KIT that is present in plasma. Dashed lines imply lanes from the same blot that were not loaded contiguously

To provide evidence that the KIT‐EV immunocapture approach presented in this study can be extended to other cell models, we applied it to the LAD2 huMC line known to recapitulate most of the characteristics and functions of primary huMC cultures. While in the neoplastic HMC‐1 cell lines KIT is expressed with gain‐of‐function mutations that keep the receptor constitutively active, LAD2 huMCs express normal KIT requiring the receptor‐ligand stem cell factor (SCF) for its activation. Fresh addition of SCF to cytokine‐starved LAD2 cells resulted in increased phosphorylation of KIT being evidence of its ligand‐mediated activation (Figure 8A). P15 and P120 EVs secreted by LAD2 cells stimulated or not with SCF contained KIT among other EV protein markers (Figure 8B). KIT‐EV immunocapture from these EV fractions resulted in the successful precipitation of KIT and other canonical EV markers and indicated that the protein composition of LAD2 KIT‐EVs changes in response to the activation of KIT by SCF (Figure 8B). These findings support the applicability of the KIT‐EV immunocapture approach to other cell models with differential KIT activity and warrant further studies on the characterization, composition, and functions of KIT‐EVs from various cell models.

### 3.8 Recovery of cell line‐derived KIT‐EVs from spiked human plasma

To validate that our immunocapture approach may also be applied to isolating KIT‐EVs from complex human biofluids, we spiked human plasma with HMC‐1.1‐derived EVs (Figure 8C). The KIT immunocapture performed from spiked plasma yielded KIT among other EV proteins (Figure 8C). Additionally, the pre‐incubation of EVs with SCF, the ligand of KIT that is also present in plasma, did not hinder the ability to immunocapture KIT‐EVs (Figure 8C). These results are indicating that our methodology can in principle be employed for the isolation of huMC EVs from plasma.

## 4 DISCUSSION

A collection of studies has underscored the heterogeneous nature of EVs released by a single cell type and emphasized the importance of the characterization of EV subpopulations that may be associated with specific biological outcomes (Lässer et al., 2018; Nikoloff et al., 2021). This is particularly important in pathological conditions where subpopulations of EVs in biological fluids may contain relevant biomarkers or serve as indicators for disease progression and/or responses to treatments (Boyiadzis & Whiteside, 2017). The receptor tyrosine kinase KIT, a mast cell signature protein, has been associated with circulating EVs from patients with systemic mastocytosis (Kim et al., 2021, 2018), acute myeloid lymphoma (Boyiadzis & Whiteside, 2017; Hong et al., 2014; Szczepanski et al., 2011), EVs released by neoplastic mast cells (Kim et al., 2018; Kim et al., 2021; Xiao et al., 2014), activated mast cells (Groot Kormelink et al., 2016; Liang et al., 2020), and GIST cells (Atay et al., 2014, 2018). Moreover, the transfer of normal and oncogenic KIT from EVs to recipient cells can alter their behaviour (Atay et al., 2014; Kim et al., 2018; Xiao et al., 2014). These findings and attributions of EVs containing KIT explain an interest in developing protocols for the specific isolation and characterization of huMC KIT‐EVs, which may have potential clinical application. In this work, we establish that KIT immunocapture is a successful approach to isolate KIT‐containing EV subpopulations secreted by huMCs, providing the groundwork for the isolation of KIT‐EVs from biological fluids in mast cell‐related diseases. We also demonstrate that KIT is a *bona* *fide* membrane component of a rather abundant subset of EVs secreted by cultured neoplastic huMCs, and that KIT‐EVs are heterogeneous regarding their physical properties, origin, and protein content, raising the possibility that distinct subtypes of mast cell KIT‐EVs target different recipient cells with specific biological effects.

By applying immunocapture of EVs using an antibody that recognizes the extracellular domain of KIT, we successfully isolated huMCs KIT‐EVs containing canonical EV protein markers, thus, providing a proof‐of‐principle that KIT‐EVs can be isolated and separated from non‐KIT‐containing vesicles. That KIT immunocapture could be developed into an established approach for isolating KIT‐EVs was further supported by the ability to immunocapture KIT‐EVs on KIT affinity EM grids. Antibody‐coated affinity grids have been described previously (Derrick, 1973; Yu et al., 2016), but to our knowledge, this is the first demonstration of capturing EV subpopulations on antibody‐coated EM grids based on the affinity to a specific EV surface component. Our immunocapture approach enabled us to define characteristics of huMC‐derived KIT‐EVs: (1) huMC EVs immunocaptured on KIT antibody‐coated affinity grids were round to oval‐shaped and heterogeneous in size, as also determined by NTA; (2) Encouraged by the MISEV2018 guidelines (Théry et al., 2018), we addressed the predicted topology of KIT within vesicle membranes, and only an antibody raised against the N‐terminal extracellular domain of KIT efficiently precipitated EVs; (3) KIT‐containing EVs represented a substantial proportion (about 40%) of total vesicles released by neoplastic huMCs. This is remarkable to note since neoplastic HMC‐1 mast cell lines carry constitutively active KIT variants (Sundström et al., 2003), similar to clonal mast cell populations in mastocytosis (Longley et al., 1999; Nagata et al., 1995; Worobec et al., 1998) and epithelial cells in GIST (Hirota et al., 1998). Oncogenic KIT shuttled in EVs may impact the pathogenesis of diseases (Atay et al., 2014; Kim et al., 2018; Kim et al., 2021), or reflect disease status as implied in studies of EGFR‐containing EVs in lung cancer or other malignancies (Bijnsdorp et al., 2021; Frawley & Piskareva, 2020; Purcell et al., 2021). Additionally, we demonstrated that KIT‐EVs can also be captured from EVs secreted by LAD2 huMCs that express normal KIT. We foresee that the KIT‐EV immunocapture methodology will also be applicable to other cell models where KIT signalling is pivotal.

This study demonstrates that KIT is incorporated into heterogeneous EVs, all of which represent relatively small vesicles (100–200 nm by NTA) of mostly low density. By applying differential ultracentrifugation, KIT‐EVs were shown to exist at least as microvesicle‐ and exosome‐like EVs bearing subtype‐characteristic protein markers. P15/microvesicle‐like KIT‐EVs were enriched for annexin A1, β‐actin, HSP90, ARF6, multiple actin cytoskeleton proteins, small G‐proteins and regulators, consistent with components involved in the biogenesis of microvesicles. On the other hand, P120/exosome‐like KIT‐EVs were enriched for CD81, ALIX, syntenin‐1, and TSG101, consistent with an endosomal origin of these vesicles.

Interestingly, the unique identity of total KIT‐EVs compared to CD9‐captured, KIT‐depleted vesicles appeared to be especially conferred by the protein profile of P15 KIT‐EVs. This profile included the above‐mentioned proteins, and numerous receptor types besides KIT (such as GPCRs, cytokine receptors and cell adhesion receptors), signalling protein and lipid kinases, phosphatases, phospholipases, adaptor proteins, ion channels and molecule transporters found within huMCs. It is worth noting that many of these proteins form signalling complexes or participate in the signalling of the receptors found in the KIT‐EVs. For example, KIT and many of its signalling components were represented, including N‐, K‐ and R‐RAS, the adaptors GRB2, LAT, SRC protein kinases (FGR, YES, LYN) and their regulators (CSK, PAG1), and others such as MAP4K4, JAK, STAT5, and STAT3. Such a combination of proteins may allow the assembly of signalosomes with activating or transforming potential in target cells. Additionally, cell‐to‐cell adhesion receptors enriched in P15 KIT‐EVs included integrins, proteins of epithelial and endothelial cell adherence and tight junctions, some of which are involved in leukocyte trans‐endothelial migration (F11R, JAM3, CD99, ITGB1, ICAM) (see Table 1).

The proteomic analysis of KIT‐EVs and P15 KIT‐EVs also offers some insight into the complex trafficking of KIT in neoplastic huMCs. Like other tyrosine kinase receptors, KIT is synthetized in the ER, matures in the Golgi and travels through vesicles to the plasma membrane, where KIT is activated upon binding to its ligand, which triggers endocytosis of the receptor (Cruse et al., 2015; Mukherjee et al., 2006). KIT continues to signal within endocytic vesicles until it is either sorted into lysosomes for degradation or recycled back to the plasma membrane. Oncogenic KIT, unlike normal KIT, is not abundant on the cell surface, but accumulates in endosomal compartments (Obata et al., 2014; Shi et al., 2016). Due to its constitutively active conformation, oncogenic KIT is rapidly internalized via clathrin‐coated vesicles when it reaches the plasma membrane (Obata et al., 2014). Also, the degradation of oncogenic KIT in lysosomes is slower than for normal KIT due to altered glycosylation patterns and defective ubiquitination (Obata et al., 2014). In this context, our data suggest KIT may be extensively routed to MVBs to be released via exosomes or through recycling endocytic vesicles to the plasma membrane to be incorporated into microvesicles, likely compensating for its defective lysosomal degradation. Supporting the concept that the trafficking of oncogenic KIT is rigorously regulated, KIT was partly diverted to microvesicle‐like EVs when the biogenesis of exosomes was reduced by GW4869 treatment, and, conversely, KIT incorporation shifted to exosome‐like EVs when the formation of microvesicles was reduced by amitriptyline. However, the biogenesis and protein sorting of KIT‐EVs may also be regulated by SMase‐independent pathways (Mathieu et al., 2019). Our proteomic data further support the abundant endocytic trafficking of KIT since KIT‐EVs were enriched for markers of recycling endosomes along with markers of MVBs, while P15 KIT‐EVs contained markers of recycling endosomes and proteins involved in the formation of clathrin‐coated pits. These included small GTPases of the RAB superfamily, which regulate multiple aspects of docking, fusion, and transport of vesicles in various vesicle compartments. Notably, RAB5, RAB2A, RAB7 and RAB11, considered markers of early endosomes, late endosomes and multivesicular bodies were enriched in KIT‐EVs, while RAB8, RAB11B, RAB13 and RAB35, markers of recycling endosomes, were enriched in P15 KIT‐EVs (Jin et al., 2021; Zerial & McBride, 2001). Thus, the different biogenesis pathways of KIT‐EVs may reflect extensive endocytic recycling of oncogenic KIT, a situation that might be recapitulated in normal cells after ligand‐induced activation, as endocytic trafficking is critical for the regulation of receptor localization, downstream signalling, as well as the duration and the extent of their activity.

Since KIT‐EVs contain distinct components, it is not surprising that a functional analysis by GO categorization of the proteins enriched in KIT‐EVs (compared to KIT‐depleted EVs), and more so in P15 KIT‐EVs (compared to P120 KIT‐EVs), indicated an association with the biological function terms “immune cell activation” and “cell migration/locomotion.” It is, thus, plausible that P15 and P120 KIT‐EVs may target different cells and initiate different biological responses – a hypothesis that will need further investigation. Although many studies have focused on exosomes, our data suggest that the microvesicle‐like P15 KIT‐EVs have unique characteristics and contain distinctive recognition and signalling proteins with the potential of modifying recipient cell behaviour. These findings should also be taken into consideration for the identification of biomarkers as many efforts to develop clinical biomarkers centre around exosomes. Resolving the heterogeneity of released EVs and increasing the understanding of the specifics of vesicle subtypes and subpopulations will lead to a more detailed understanding of unique versus overlapping biological functions of EVs, or beneficial versus detrimental effects of subpopulations, which is especially relevant for the development of EV‐based therapeutical approaches (Lässer et al., 2018).

Considering that virtually any cell type can release EVs, the heterogeneity of EVs in biological samples is complex and the EV donor sources are largely indistinguishable. Efforts have been made to determine surface markers that allow the enrichment of cell type or cell state‐specific EVs from biofluids (Melo et al., 2015; Mustapic et al., 2017; Sharma et al., 2018). We are, therefore, advocates of the application of KIT immunocapture as an approach for enriching huMC‐specific EVs from complex biological samples such as patient plasma. Our EV immunocapture results from spiked human plasma indicated that a recovery of huMC‐derived KIT‐EVs from plasma is in principle possible. We are, thus, in the process of determining the experimental details of isolating in vivo KIT‐EVs from bioliquids. Following this approach will provide significant insight into the cargo of in vivo huMC EVs and the opportunity to compare the content of huMC EVs under normal and diseased conditions, with a potential use as biomarkers of specific pathology, as also proposed in a study analyzing KIT‐containing exosomes from patients with GIST before and after treatment with tryosine kinase inhibitors (Atay et al., 2018).

In summary, this study describes the successful separation of KIT‐EVs by selective immunocapture from vesicles shed by cultured neoplastic huMCs. This applied methodology will potentially enable the enrichment of KIT‐EVs from complex biological samples. Our thorough characterization of huMC‐derived KIT‐EVs underlines the heterogeneity of KIT‐EVs in terms of biogenesis, composition, and properties, thus, raising the possibility that distinct subtypes of KIT‐EVs target different recipient cells with potentially distinct biological effects, and that further study of their specific cargo in mast cell‐related or other diseases may yield potential biomarkers or read‐outs of disease.

## AUTHOR CONTRIBUTIONS

Annika Pfeiffer: Conceptualization; Data curation; Formal analysis; Investigation; Methodology; Project administration; Validation; Visualization; Writing – original draft; Writing – review & editing. Jennifer D. Petersen: Data curation‐Equal; Formal analysis‐Equal; Investigation‐Equal; Methodology‐Equal; Writing – review & editing‐Equal. Guido H. Falduto: Investigation‐Equal; Methodology‐Equal. David Eric Anderson: Conceptualization‐Supporting; Data curation‐Supporting; Formal analysis‐Supporting; Methodology‐Equal. Joshua Zimmerberg: Funding acquisition; Resources; Writing – review & editing. Dean D. Metcalfe: Funding acquisition‐Lead; Resources‐Equal; Supervision‐Supporting; Writing – review & editing‐Supporting. Ana Olivera: Conceptualization; Formal analysis; Funding acquisition; Project administration; Resources; Supervision; Visualization; Writing – review & editing.

## CONFLICT OF INTEREST

The authors report no conflict of interest.

## Supporting information


**Supplementary Figure 1**. Comparison of ExoQuick with 12% and 8% PEG solutions.
**Supplementary Figure 2**. Assessment of the topology of KIT within EV membranes.
**Supplementary Figure 3**. Preparation and specificity of KIT‐EV immunocapture on affinity EM grids.
**Supplementary Figure 4**. Comparison of KIT‐positive versus KIT‐negative EVs.
**Supplementary Figure 5**. Normalized proteomics data. (A) Hierarchical clustering of normalized log2 H/L ratios obtained from the SILAC analysis of KIT‐containing versus KIT‐depleted EVs.
**Supplementary Figure 6**. GO analysis of significantly enriched proteins in KIT(+) EVs.

**Supplementary Table I** ‐ Enriched proteins in KIT‐EVs (compared to KIT(‐)/CD9(+) EVs) or in P15 KIT‐EVs (compared to P120 KIT‐EVs) grouped by functional categories that were defined by high‐level GO “biological process” terms.

Supplementary Information ‐ Comparison of proteins present in huMC‐derived KIT‐EVs (Pfeiffer et al., this study) and GIST exosomes (Atay et al., 2018).

Supplementary information
