# Supplementary material for: Selective immunocapture reveals neoplastic human mast cells secrete distinct microvesicle‐ and exosome‐like populations of KIT‐containing extracellular vesicles
Source: J Extracell Vesicles. 2022 Oct 14;11(10):12272. doi: 10.1002/jev2.12272 (PMC9838129; doi:10.1002/jev2.12272)
Supplement: Supplementary file 1 — Supplementary Figure 1. Comparison of ExoQuick with 12% and 8% PEG solutions. Supplementary Figure 2. Assessment of the topology of KIT within EV membranes. Supplementary Figure 3. Preparation and specificity of KIT‐EV immunocapture on affinity EM grids. Supplementary Figure 4. Comparison of KIT‐positive versus KIT‐negative EVs. Supplementary Figure 5. Normalized proteomics data. (A) Hierarchical clustering of normalized log2 H/L ratios obtained from the SILAC analysis of KIT‐containing versus KIT‐depleted EVs. Supplementary Figure 6. GO analysis of significantly enriched proteins in KIT(+) EVs. [file JEV2-11-12272-s001.docx]

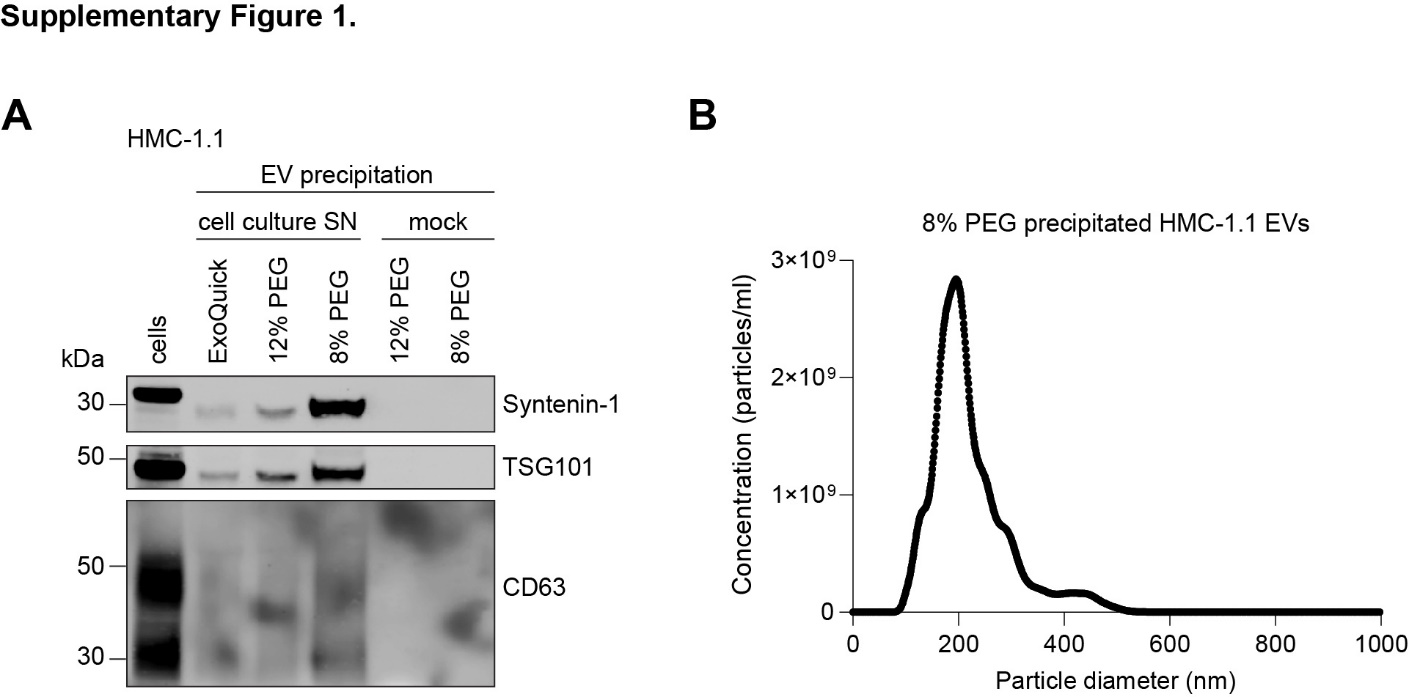


Supplementary Figure 1. **Comparison of ExoQuick with 12% and 8% PEG solutions.** (A) EVs were precipitated by ExoQuick, 12% or 8% PEG from HMC-1.1 cell culture supernatant (SN) or from full media not exposed to cells (mock). Cell and EV lysates were analyzed by immunoblotting with the indicated antibodies. (B) Nanoparticle tracking of HMC-1.1-derived EVs precipitated by 8% PEG. Representative graph showing the averaged profile of five workflow repetitions.


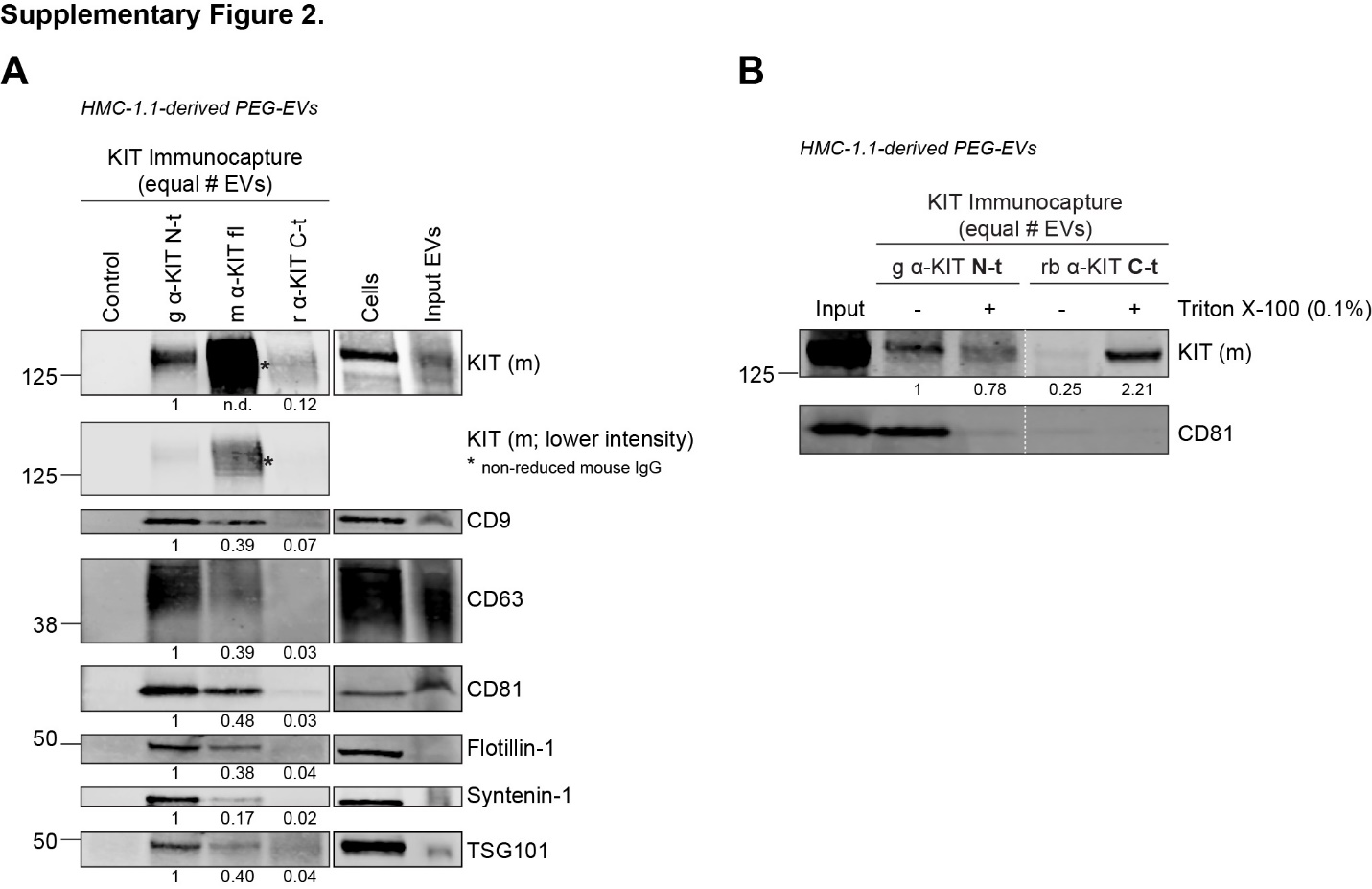


Supplementary Figure 2. **Assessment of the topology of KIT within EV membranes.** (A) Representative immunoblot of KIT-EV immunocapturing. Three different antibodies directed against N-terminal (N-t), full-length (fl) or C-terminal (C-t) KIT protein were used for the immunocapturing of KIT-containing EVs. Each immunocapture was performed from an equal number of vesicles (3x10^10^ EVs/immunocapture) and total eluates were loaded. Unconjugated beads served as a negative control. Cell and input EV lysates (30 µg) are shown on the right. The canonical EV marker content was assessed by Western blotting and quantified using the Image Studio Lite software. Indicated below each blot is the fold change in signal intensity compared to EVs captured with anti-KIT (N-terminal) antibody. *, signal from non-reduced mouse IgG over-shadowing the signal for KIT since both the capturing (m anti-KIT fl) and the detection KIT antibodies were from mouse. A lower intensity blot is included for the detection of KIT to show the broad band resulting from mouse IgG. g, goat; m, mouse; rb, rabbit. N.d., not determined. (B) HMC-1.1-derived PEG-precipitated EVs (3.8x10^9^ EVs/immunocapture) were lysed or not with 0.1% Triton X-100 detergent, followed by KIT-EV immunocapturing with two different antibodies and immunoblotting of the total eluates. Input EVs (30 µg) are shown in the first lane. The absence of co-precipitated CD81 with the KIT capture in Triton X-100-treated samples supports the EV lysis efficiency.


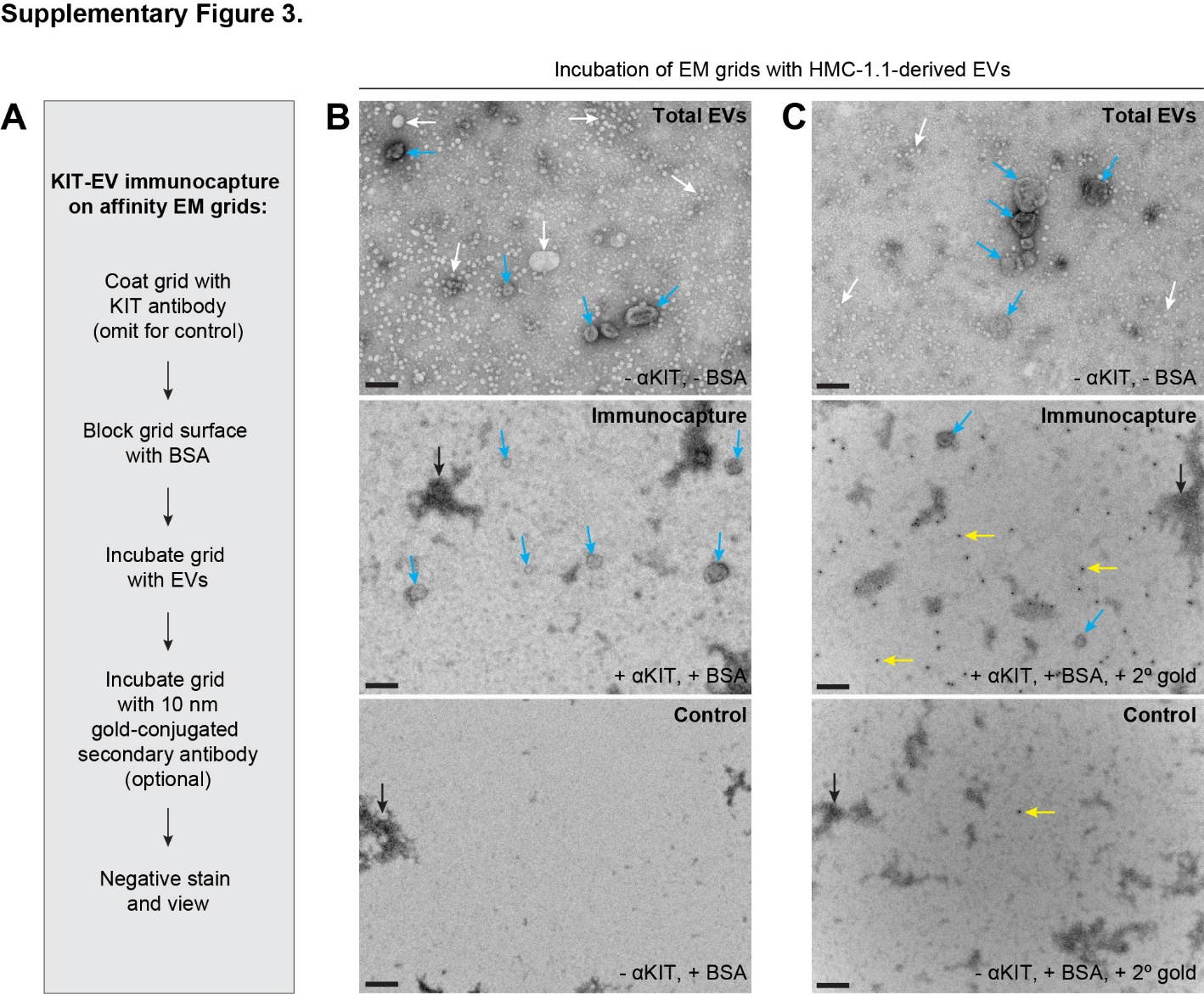


Supplementary Figure 3. **Preparation and specificity of KIT-EV immunocapture on affinity EM grids.** (A) The experimental steps for negative stain EM of immunocaptured KIT-EVs on antibody-coated affinity grids (box). (B, C) Examples from two replicates of PEG-precipitated HMC-1.1-derived EVs adhered to regular EM grids (Total EVs, top), KIT-EVs immunocaptured on affinity grids (Immunocapture, middle) or control grids (Control, bottom) viewed by negative stain EM. ‘Total EVs’ showed EV-like cup-shaped vesicles bound to the grid surface (blue arrows). Numerous lipoprotein-like non-membrane-bound particles (white arrows) were also present. Immunocapture grids (middle) displayed membrane-bound vesicles, while the non-membrane-bound particles were excluded. A fuzzy material was present (black arrows) after incubation of grids with antibody and BSA blocking solution. Control grids (bottom) in which the KIT antibody was omitted, and the grid surface was blocked with BSA before incubation with the EVs exhibited virtually no bound vesicles or lipoprotein-like entities. (C) In this example, the optional step of incubating the grid on 10 nm gold-conjugated secondary antibody (yellow arrows) was included to demonstrate the presence of primary antibody on the affinity grid surface, and its nearly complete absence on the control grid that lacked the KIT primary antibody. All scale bars are 200 nm.


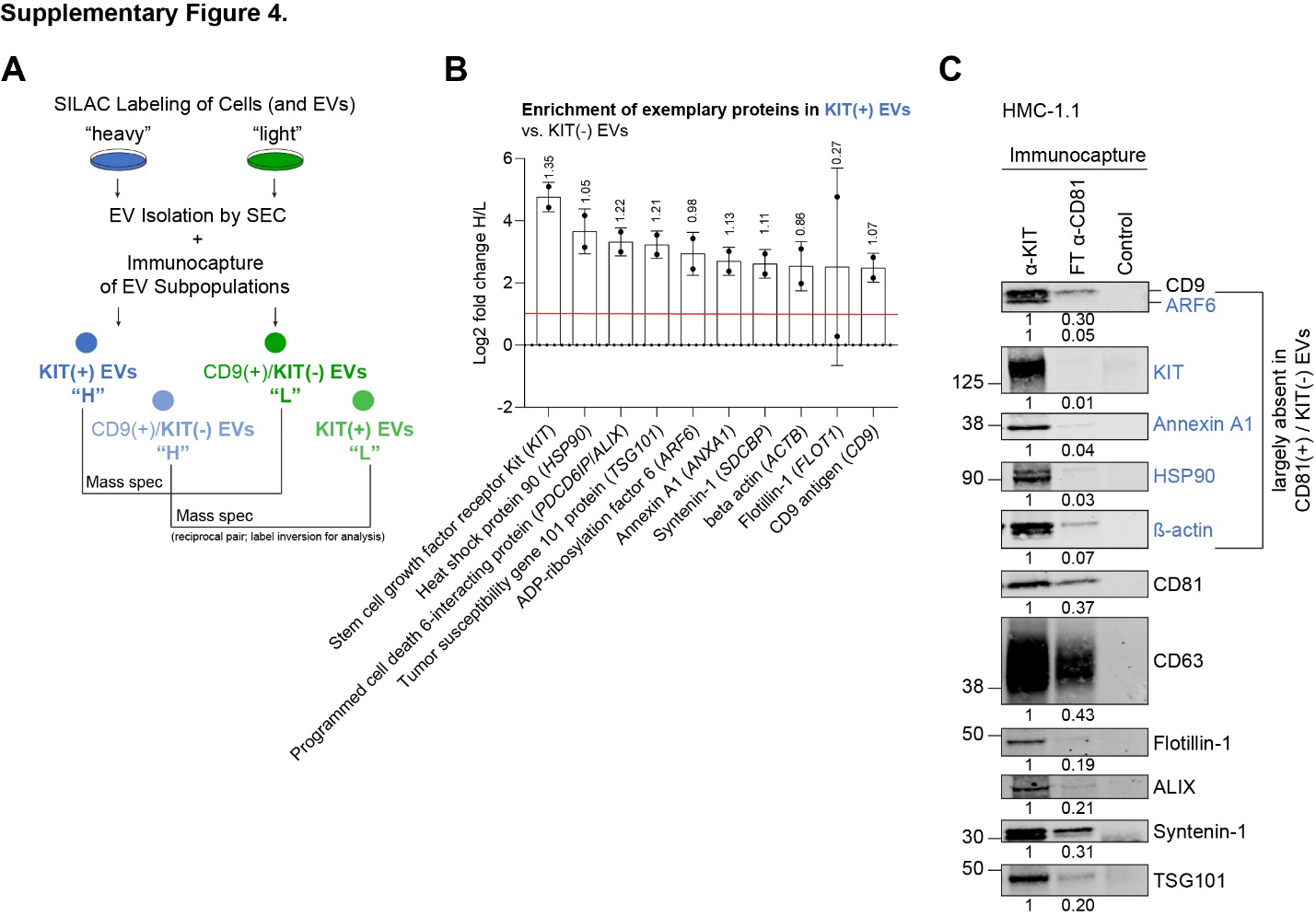


Supplementary Figure 4. **Comparison of KIT-positive versus KIT-negative EVs.** (A) HMC-1.1 cells were SILAC-labeled for performing quantitative mass spectrometry on cell-released EVs. Vesicles were isolated by size exclusion chromatography (SEC) and KIT-containing EVs were captured by immunoaffinity. Heavy-labeled KIT(+) EVs were mixed with light-labeled KIT-depleted EVs, and vice versa, and processed for mass spectrometry. (B) The enrichment of exemplary proteins in KIT-containing versus KIT-depleted EVs (presented as log2 fold change H/L determined by SILAC mass spectrometry) is shown. Data are presented as the mean from two mass spectrometry pairs ± S.D. The red line indicates the 2-fold enrichment threshold. Gene names are shown in italic. The -Log T-test p-value is shown above each bar. -Log T-test p-values >1.3 indicate significance. (C) As in Figure 3e, HMC-1.1-derived EVs were exposed to KIT immunocapture. The KIT-depleted flow-through (FT) was consecutively incubated with CD81-conjugated beads to isolate KIT-negative/CD81-positive EVs. Unconjugated beads served as a negative control. EV lysates were tested by immunoblotting with the indicated antibodies. Proteins largely absent in KIT-negative/CD81-positive EVs are highlighted in blue.


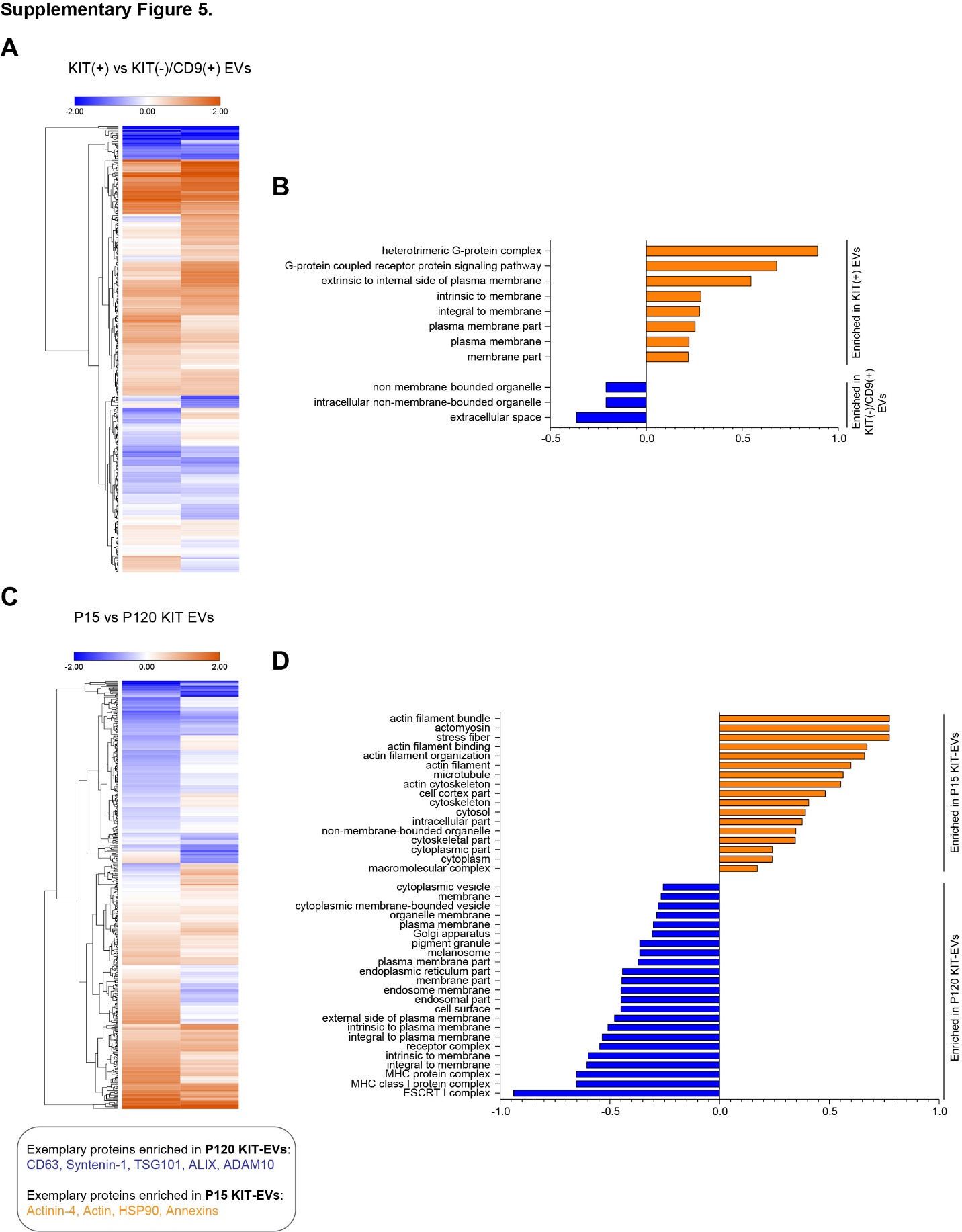


Supplementary Figure 5. **Normalized proteomics data.** (A) Hierarchical clustering of normalized log2 H/L ratios obtained from the SILAC analysis of KIT-containing versus KIT-depleted EVs. Each column of the heat map represents a replicate. (B) 1D GO analysis of proteins (A) upregulated in KIT(+) EVs (orange) or in KIT(-)/CD9(+) EVs (blue). (C) Hierarchical clustering of normalized log2 H/L ratios obtained from the SILAC analysis of P15 versus P120 KIT-EVs. Each column of the heat map represents a replicate. Exemplary proteins enriched in either P120 or P15 KIT-EVs are highlighted (box). These proteins have also been previously associated with exosome-like (P120) or microvesicle-like (P15) EVs. (D) 1D GO analysis of proteins (C) upregulated in P15 KIT-EVs (orange) or in P120 KIT-EVs (blue).


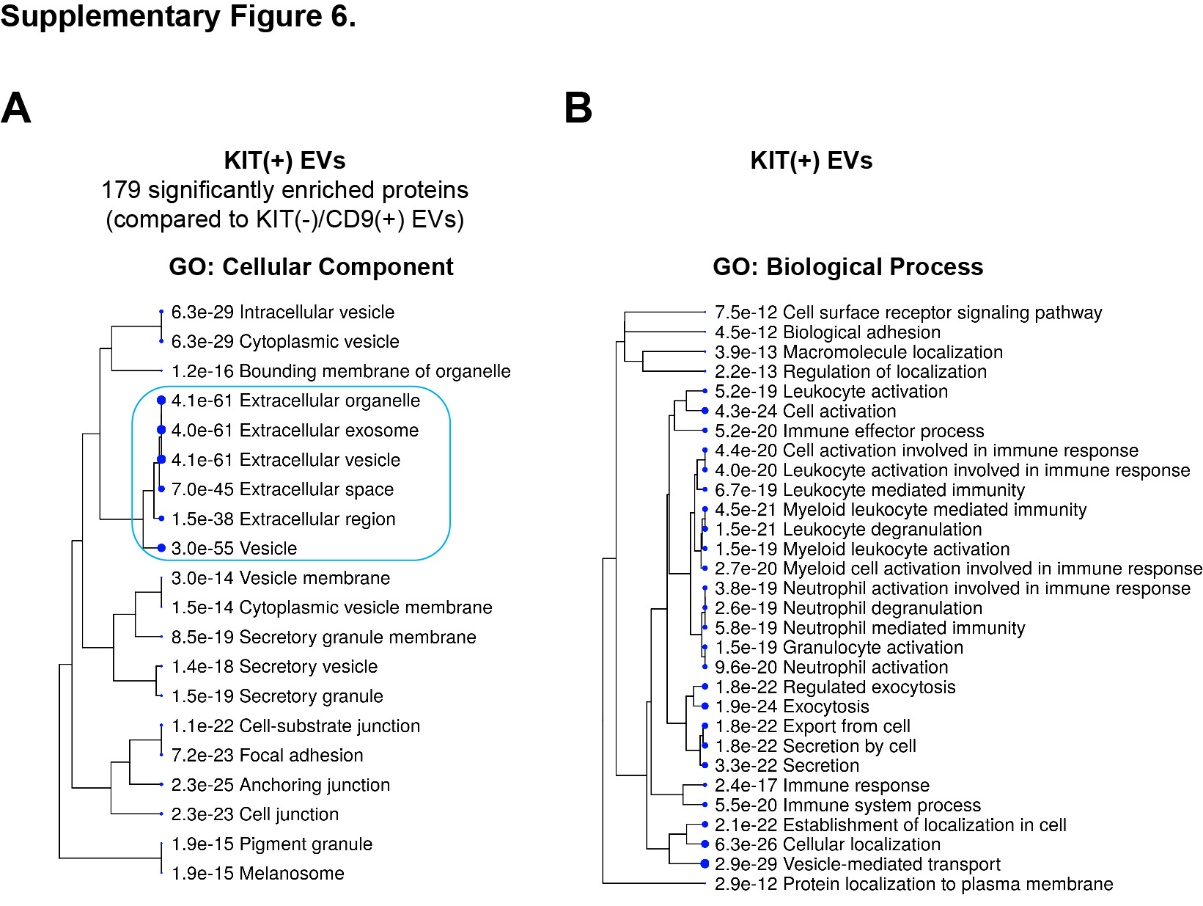


Supplementary Figure 6. **GO analysis of significantly enriched proteins in KIT(+) EVs.** (A) Significantly enriched proteins in KIT(+) EVs were analyzed for GO enrichment of “Cellular Component” categories. The top 20 pathways are shown in a hierarchical clustering tree graph that is indicating the statistical significance by dot size. (B) Significantly enriched proteins in KIT(+) EVs were analyzed for GO enrichment of “Biological Process” terms. The top 30 hits are displayed in a hierarchical clustering tree graph and the statistical significance is indicated by the size of the dots.
