# Supplementary material for: Selective immunocapture reveals neoplastic human mast cells secrete distinct microvesicle‐ and exosome‐like populations of KIT‐containing extracellular vesicles
Source: J Extracell Vesicles. 2022 Oct 14;11(10):12272. doi: 10.1002/jev2.12272 (PMC9838129; doi:10.1002/jev2.12272)
Supplement: Supplementary file 2 — Supplementary Table I ‐ Enriched proteins in KIT‐EVs (compared to KIT(‐)/CD9(+) EVs) or in P15 KIT‐EVs (compared to P120 KIT‐EVs) grouped by functional categories that were defined by high‐level GO “biological process” terms. [file JEV2-11-12272-s002.docx]

**Supplementary Table I –** Enriched proteins in KIT-EVs (compared to KIT(-)/CD9(+) EVs) or in P15 KIT-EVs (compared to P120 KIT-EVs) grouped by functional categories that were defined by high-level GO “biological process” terms.

| **Enrichment in KIT-EVs (vs. KIT(-)/CD9(+) EVs)** | | | **Enrichment in P15 KIT-EVs (vs. P120 KIT-EVs)** | | |
| --- | --- | --- | --- | --- | --- |
| **N** | **High level GO Category** | **Proteins** | **N** | **High level GO Category** | **Proteins** |
| 94 | *Cellular localization* | CCT7 EHD2 RAB7A STXBP2 RAB10 SNAP23 RAB5C RAB14 VPS25 EFR3A SEPTIN11 MVB12A CALM1 GOLGA7 RAB8A ARF4 KPNA2 TRPV2 ANXA6 MYO1C SNX2 VPS28 ATP2B4 ATP1A1 FLNA CSK YKT6 CD81 VPS4B ITGB1 AP2B1 BTK CDC42 EZR MYL12A ITGA4 ESYT1 IQGAP1 CLTC PAK1 ZDHHC5 PRKCB BSG CIB1 RAB11B LAT TXNIP RDX TFRC EPB41L2 NUDC DIAPH1 TMBIM1 TNIK KIT F11R CTTN RFTN1 RALB MARK2 GNAS ITGA2B PLAUR CD44 GDI2 SLC2A3 FCGR2B CD59 RPLP0 RAB2A SIGLEC5 PDAP1 VAT1 ALDOC CAND1 ADGRE5 RAP2C ABCC4 OSTF1 ITGAV FCGR2A IQGAP2 RPL7 ENDOD1 CSTB NCSTN S100A11 RPSA RAP2B SIRPA NRAS CPNE1 PLEKHO2 LAIR1 | 116 | *Immune system process* | FGR CD99 FCGR2B PAG1 CD59 LAT2 CSK NECTIN2 FCGR2A CYRIB KIT SYK YES1 ARHG CADM1 SPN LAT LYN TNFRSF14 IL6ST ITGB1 CD44 TESC PYCARD RPS19 F11R JAM3 STAT3 ITGA2B WAS RHOA CDC42 ICAM1 JAG1 STX4 ZC3HAV1 RAP1A MPP1 DBNL PIP4K2A ENPP3 EPHB1 WASF2 ITGA2 OTUB1 GCSAML OXSR1 CD151 CDC42EP4 MYO1C HLA-B NME1 IL10RB LAIR1 PLEK ADD1 FKBP12-Exip3 SNAP23 RASAL3 RAB35 RRAS RFTN1 NCK1 BSG RAB6A DOCK10 PRKCA PLAUR HEL114 SLC2A3 ATP1B3 DYNC1I2 GNB1 PGM1 GNAS HEL-S-276 COTL1 SLC7A5 TYK2 KPNB1 VAT1 CAND1 TMEM30A ADGRE5 RAP1B KRAS TMBIM1 CAB39 KIF23 ITGAV TMC6 PSMA5 GOLGA7 PSMC2 ITGA5 RACGAP1 JAK1 S100A11 ANPEP HEL-S-1 SLC3A2 RAB31 PA2G4 PGAM1 HSPA6 GRB2 RAP2B IST1 GNG2 FLNA IGF2R SVIP NRAS PLEKHO2 SLC16A1 PSME2 |
| 84 | *Immune system process* | FCGR2B PAG1 CD59 GGT1 CSK OAS2 FCGR2A KIT YES1 CADM1 IFITM2 SIRPA LAT ITGA4 ITGB1 BTK CD44 TFRC CD81 F11R ITGA2B CDC42 EFNB1 FLNB DOCK11 WASF2 NCSTN ITGA2 PRKCB GCSAML CIB1 PLSCR1 MYO1C HLA-Cw HLA-A NME1 RHEX LAIR1 INPP5D STXBP2 RAB10 SNAP23 EZR RFTN1 BSG AP2B1 PLAUR GDI2 SLC2A3 RAB7A GNB1 GNAS SIGLEC5 PDAP1 RAB5C VAT1 ALDOC CAND1 RAB14 ADGRE5 RAP2C DIAPH1 KRAS OSTF1 TMBIM1 ITGAV IQGAP1 CLTC SLC16A3 IQGAP2 GOLGA7 PAK1 CSTB ITGA5 S100A11 SLC3A2 RAP2B FLNA L1CAM NRAS CPNE1 PLEKHO2 SLC16A1 PSME1 | 110 | *Regulation of biological quality* | HEL114 PLEK FLNA ATP2B4 RHOA ATP1B3 ATP2B1 ADD1 GNA11 SNAP23 COTL1 STX4 TMEM30A RAP1A RAP1B DBNL ARPC5L ARHGAP18 ADD3 WASF2 F11R ATP1A1 SYK ANPEP RAB8A ARHG CDC42EP4 FMNL1 TRPV2 PLXNB2 LYN GSTO1 GNAS FKBP12-Exip3 TESC RANGAP1 SLC7A5 YWHAH ALDH9A1 GOLGA7 PRKCA HEL-S-1 BSG RAP2B FGR ITGA2B WAS DIP2B ACSL4 CDC42 MAP4K4 GNB1 ICAM1 PYCARD CSK NCS1 EHD1 OSBP SLC29A1 GNAI2 WDR48 GNA13 AARS2 DSTN STAT5A KRAS RDX ITGAV SEPTIN11 ITGB1 CYRIB EPHB1 KIT NCK1 ANXA5 STXBP5 ARF6 JAM3 ARF4 STAT3 CLIC4 CNP GRB2 IST1 RAB11B MYO1C LAT NME1 SLC12A7 CYFIP1 FCGR2B RPS19 PDCD10 EPB41 DDB1 ABCC1 DOCK10 PLAUR EHD2 GNAI3 CD59 HEL-S-276 RENBP CAND1 PSMA5 PSMC2 ITGA2 YES1 GNG2 PSME2 |
| 73 | *Regulation of biological quality* | MYL12A FLNA PLXND1 ATP2B4 TFRC STXBP2 SNAP23 PLIN3 DKFZp761E1322 CALM1 ADD3 GNAQ WASF2 F11R ATP1A1 RAB8A TRPV2 PLSCR1 ANXA6 CTTN SCARB1 GNAS IQGAP2 GOLGA7 PLCB3 TMC8 BSG RAP2B ITGA2B AP2B1 BTK PLEKHO1 DIP2B ACSL4 CDC42 GNB1 EZR CSK OAS2 GNAI2 GNA13 KRAS RDX ITGAV SEPTIN11 DOCK11 PAK1 ITGB1 KIT NCSTN PDLIM5 PRKCB ARF4 RAB11B MYO1C LAT NME1 RHEX FCGR2B RAB7A CD81 DIAPH1 CCT7 MARK2 L1CAM PLAUR EHD2 GNAI3 CD59 CAND1 ITGA2 YES1 PSME1 | 109 | *Cellular localization* | EHD2 RANBP5 DYNC1I2 SNAP23 STX4 KPNB1 EHD1 RAB35 RAP1A RAP1B NECTIN2 REPS1 SEPTIN11 RAB13 GOLGA7 SYK ARF6 RAB8A ARF4 SEPTIN2 RAB31 RAB6A KPNA2 TRPV2 MYO1C SVIP SNX2 LYN ATP2B4 GSTO1 ATP1A1 FLNA ATP1B3 FKBP12-Exip3 TESC RANGAP1 CSK YKT6 TMEM30A ITGB1 STAT3 FGR WAS CDC42 MAP4K4 LAT2 NCS1 OSBP PLEK YWHAH ZDHHC5 STXBP5 HEL-S-1 BSG RAB11B LAT RDX HEL114 EPB41L2 SLC29A1 PDCD10 TMBIM1 PIP5K1A PIP4K2A TNIK KIT F11R EPB41 JAM3 ARHG AFDN RFTN1 ATG3 GNAS ITGA2B PLAUR CD44 SLC2A3 RHOA FCGR2B PGM1 CD59 COTL1 PYCARD RPS19 PPP2R1A VAT1 CAND1 ADGRE5 CAB39 DBNL ITGAV TMC6 PSMA5 FCGR2A CYRIB PSMC2 S100A11 ANXA5 ANPEP PA2G4 PGAM1 HSPA6 RAP2B IST1 IGF2R NRAS PLEKHO2 LAIR1 |
